# Single-cell transcriptome and antigen-immunoglobin analysis reveals the diversity of B cells in non-small cell lung cancer

**DOI:** 10.1186/s13059-020-02064-6

**Published:** 2020-06-24

**Authors:** Jian Chen, Yun Tan, Fenghuan Sun, Likun Hou, Chi Zhang, Tao Ge, Huansha Yu, Chunxiao Wu, Yuming Zhu, Liang Duan, Liang Wu, Nan Song, Liping Zhang, Wei Zhang, Di Wang, Chang Chen, Chunyan Wu, Gening Jiang, Peng Zhang

**Affiliations:** 1 Department of Thoracic Surgery, Shanghai Pulmonary Hospital, Tongji University School of Medicine, Shanghai, 200433 China; 2 National Research Center for Translational Medicine, Ruijin Hospital, Shanghai Jiaotong University School of Medicine, Shanghai, 200025 China; 3 Department of Pathology, Shanghai Pulmonary Hospital, Tongji University School of Medicine, Shanghai, 200433 China; 4 Department of Medical and Molecular Genetics, Indiana University School of Medicine, Indianapolis, IN 46202 USA; 5 Animal Laboratory Center, Shanghai Pulmonary Hospital, Tongji University School of Medicine, Shanghai, 200433 China; 6 Shanghai Municipal Center for Disease Control and Prevention, Shanghai, 200126 China

## Abstract

**Background:**

Malignant transformation and progression of cancer are driven by the co-evolution of cancer cells and their dysregulated tumor microenvironment (TME). Recent studies on immunotherapy demonstrate the efficacy in reverting the anti-tumoral function of T cells, highlighting the therapeutic potential in targeting certain cell types in TME. However, the functions of other immune cell types remain largely unexplored.

**Results:**

We conduct a single-cell RNA-seq analysis of cells isolated from tumor tissue samples of non-small cell lung cancer (NSCLC) patients, and identify subtypes of tumor-infiltrated B cells and their diverse functions in the progression of NSCLC. Flow cytometry and immunohistochemistry experiments on two independent cohorts confirm the co-existence of the two major subtypes of B cells, namely the naïve-like and plasma-like B cells. The naïve-like B cells are decreased in advanced NSCLC, and their lower level is associated with poor prognosis. Co-culture of isolated naïve-like B cells from NSCLC patients with two lung cancer cell lines demonstrate that the naïve-like B cells suppress the growth of lung cancer cells by secreting four factors negatively regulating the cell growth. We also demonstrate that the plasma-like B cells inhibit cancer cell growth in the early stage of NSCLC, but promote cell growth in the advanced stage of NSCLC. The roles of the plasma-like B cell produced immunoglobulins, and their interacting proteins in the progression of NSCLC are further validated by proteomics data.

**Conclusion:**

Our analysis reveals versatile functions of tumor-infiltrating B cells and their potential clinical implications in NSCLC.

## Introduction

Non-small cell lung cancer (NSCLC) is the leading life-threatening cancer in the world [1, 2]. With the advancement in surgery, radiotherapy, chemotherapy, and immunotherapy, the prognosis of NSCLC has been significantly improved [3], but the clinical outcome of advanced-stage NSCLC remains unsatisfied. Recent studies reported the responsiveness of immunotherapy is highly determined by the characteristics of tumor microenvironment (TME), the variations in which were manifested by different abundance and functions of tumor-infiltrating lymphocytes (TIL), myeloid, and other stromal cells. A systematic characterization of the landscape of the TME and the crosstalk between different cell types lays the biological foundations for optimizing personalized immunotherapies of NSCLC.

T cells and B cells are the most abundant lymphocyte populations and play pivotal roles in the TME of solid tumors. While T cells have been widely studied and therapeutically targeted in immunotherapy, unfortunately, currently approved immune checkpoint inhibitors only achieved 20–25% response rate in unscreened NSCLC [4–8]. Tumor-infiltrating B cells are a key component of adaptive immunity with diverse functions. Inconsistent anti-tumor effects of B cells in NSCLC have been reported [9]. The subtypes and mechanisms of B cells in the TME of NSCLC and how they interact with cancer cells and other stromal cell types remain largely unknown. Hence, a comprehensive characterization of the NSCLC immuno-landscape including the distribution and role of immune cell subtypes, especially B lymphocytes, is necessary for understanding the non-responsiveness mechanisms and discovering novel biomarkers and therapeutic strategies.

B cell regulates immune responses and inflammation through antibody production and inducing T cell activation and proliferation via antigen presentation [10, 11]. Recent studies revealed that depletion of B cells using anti-IgM antibodies reduced tumor burden in mouse, indicating a possible role of B cells in regulating cancer cells’ progression [12]. Indeed, several studies identified that the B cells activated the FcRγ receptors on myeloid cells and further induced carcinogenesis of squamous cells [13]. Another study revealed that B cells may promote metastasis of breast cancer via secretion of HSPA4-targeting immunoglobins (IgG) via activating the CXCR4/SDF1α axis in tumor cells [14]. Contrarily, several studies unveiled potential anti-tumor functions of B cells. Depletion of B cells using antibodies against CD20 enhanced the progression of melanoma in mouse [15]. Activation of B cells can also increase the T cell-mediated anti-tumor effects and has been directly utilized in complement-mediated tumor cell lysis through the production of IgM, IgG, and IgG2b [16]. Noting the different B cell subtypes and their interactions with other cell types may cause such varied double-edge effects, it is necessary to characterize B cells and other immune cell types in the TME at single-cell resolution.

Single-cell transcriptome and data analysis enables a comprehensive characterization of the cell types in TME with high resolution [17, 18]. Until 2020, single cell-based characterization of immune landscape, transcriptome signature, and varied functions of immune cells has been conducted for breast cancer [17], lung cancer [19], liver cancer [20], colorectal cancer [21], melanoma [22], and head and neck cancer [23]. However, those studies majorly focused on T cells [17–20] or stromal cells [24, 25], but the distinct subtypes of B cells, as well as their functions, were largely ignored.

In this study, we conducted a single-cell RNA-seq analysis of 115,545 cells in 11 NSCLC patients, to systematically examine the relation between tumor-infiltrating B cell profiles and the clinical outcome of NSCLC patients. Our analysis identified the co-existence of two classes of B cells in the TME of NSCLC. We further validated the existence and determined the clinical impact of these two B cell subtypes by using immunohistochemistry and flow cytometry in two cohorts. Also, we performed functional analyses to identify the mechanisms of the two classes of B cells in the progression of NSCLC. Collectively, our study not only included a high-quality single-cell transcriptomic reference map of the TME of stage I–III NSCLC, but also provided mechanistic explanations to the role of B cells in NSCLC and implicated promising therapeutic potentials.

## Results

### Infiltration of immune cells in NSCLC

**Fig. 1:**
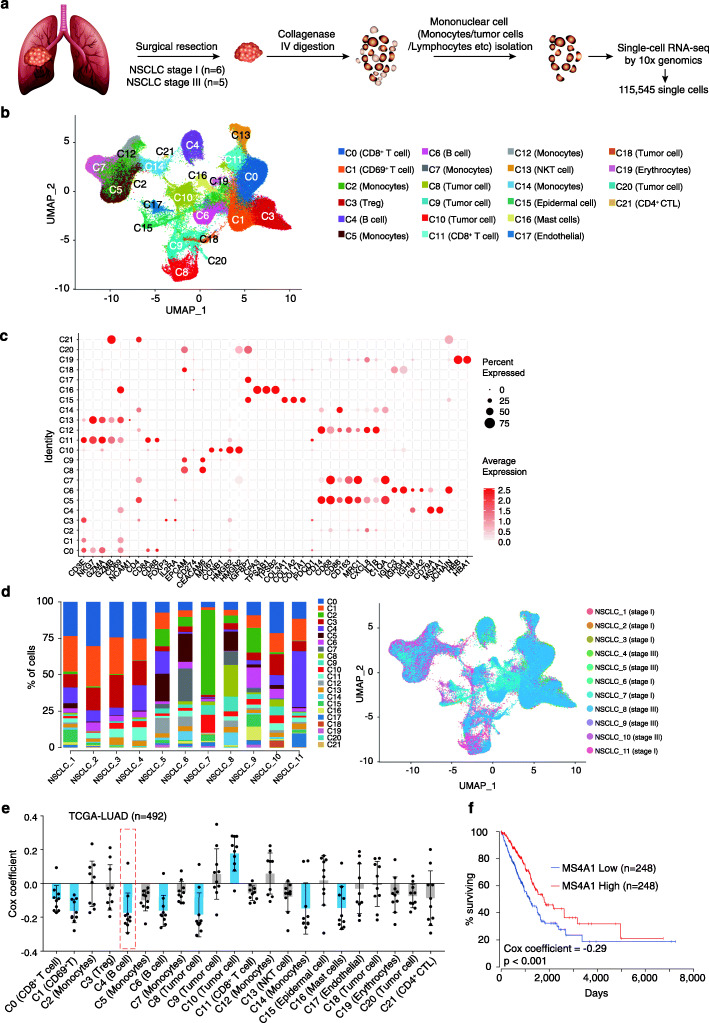
Single-cell transcriptomic analysis reveals the transcriptome of cells in the microenvironment of lung cancer. **a** Schematic illustration of the experimental design of this study. **b** Visualization of single-cell RNA-seq data of 115,545 cells freshly isolated primary lung cancer by UMAP. **c** Representative gene expression in 22 subclasses of cells. **d** Distribution of the 22 subclasses among 11 patients with NSCLC. **e**, **f** The prognosis value of the genes uniquely expressed in each class of the 22 cell types

To systematically investigate the TME of the NSCLC, we performed single-cell RNA-seq analysis using the fresh tumor samples collected immediately after surgery. Tumor tissues from 11 patients with NSCLC, including 6 of stage I and 5 of stage III, were digested with the collagenase IV and analyzed by scRNA-seq with a total of 115,545 single cells (Fig. 1a), while the red blood cells, the platelets, and the dead cells were removed by gradient centrifuge with the Ficoll [26]. Detailed data processing procedures are available in the “Methods” part. We merged the single-cell transcriptomic profiles collected from different samples for a UMAP-based cell clustering analysis, the cell types of which were further annotated by using known cell type-specific gene markers [27]. In total, 22 distinct cell clusters were identified and annotated, including subtypes of T cells, B cells, monocytes, tumor, and epidermal cells (Fig. 1b).

We identified six classes of T cells (C0, C1, C3, C11, C13, C21) (Fig. 1b, Additional file 1: Figure S1, Additional file2: Table S1). The C0 and C11 cluster of T cells are with high expression level of CD3E, CD8A, and CD8B, suggesting CD8^+^ T cells; the C13 cluster of T cells expressed high level of NCAM1 (CD56), CD3E, and NKG7 and low level of CD8A and CD8B, suggesting possible natural killer T (NKT) cells; the C1 cluster expressed CD3E and CD69 genes, indicating CD69^+^ T cells; the C21 expressed high levels of CD4 and GZMB, indicating CD4^+^ CTLs; the C3 cluster is with high expression of FOXP3 and IL2RA (CD25), which are possible Tregs. We identified two classes of B cell (C4, C6), which expressed CD79A (in C4 and C6) and MS4A1 (CD20) (in C4). We identified five classes of tumor cells (C8, C9, C10, C18, C20), which expressed the tumor cell marker genes EPCAM, CEACAM6, or MKI67. We also identified five classes of monocytes (C2, C5, C7, C12, C14), expressing CD14 and CD86. The C2, C5, C7, and C14 expressed high levels of CD163 and MRC1 (CD206), suggesting the majority of monocytes/macrophages in the TME are M2-polarization macrophage. We also observed red blood cell (C19) expressing HBA1 and HBB, epidermal or vascular cells (C15 and C17) expressing COL3A1 and COL1A2 (in C15) or IGFBP7 (in C17), and mast cells (C16) expressing TPSAB1 and TPSB2 in the scRNA-seq (Fig. 1c, Additional file 1: Figure S1, Additional file 2: Table S1). We next detected the ratio of 22 types of cells in different patients. In accordance with previous observations [17], the composition of each cell types was largely different across the 11 tumor samples (Fig. 1d).

To identify the clinical impact of these cell types in NSCLC, we selected the top 20 genes that mostly determined the identity of each cell type by ROC analysis. Co-relation between the expression level of these genes and the prognosis of the NSCLC was further computed by multivariate Cox regressions in TCGA data (Additional file 3: Table S2). We found that the genes exclusively expressed in C10 (tumor cell) (ave.cor = 0.178) are associated with poor prognosis (Fig. 1e), where ave.cor represents the averaged Pearson correlation coefficient. On the other hand, the C1 (CD69^+^ T cell) (ave.cor = − 0.164) and C4 (B cell) (ave.cor = − 0.174) expressing genes are correlated with favorable prognosis of NSCLC, implying possible tumor-suppressive functions of these cells. It is noteworthy that the higher expression of the genes exclusively expressed in the C4 B cell types, such as MS4A1 (also known as CD20 antigen), were positively with favorable prognosis of NSCLC (Fig. 1e, f), suggesting a tumor-suppressive function of C4 cells in the microenvironment of NSCLC. Noting the significant association with prognosis, we further focused on the subtypes of B cells and their biological characteristics in the TME of NSCLC.

### Co-existence of multiple subclasses of B cells in NSCLC

**Fig. 2:**
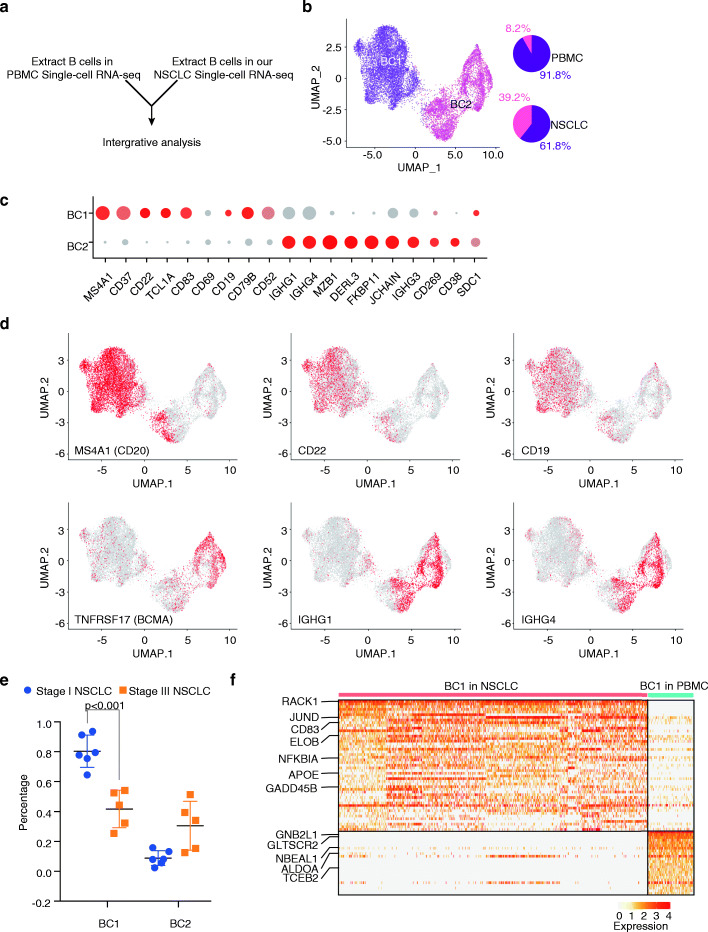
Co-existence of the two major subtypes of B cells in the microenvironment of NSCLC tumors. **a** Schematic illustration of the analysis procedure. **b** Visualization of single-cell RNA-seq data of B cells by UMAP. **c** Representative gene expression among different classes of B cells. **d** Expression of MS4A1 (CD20), CD22, CD19, TNFRSF17 (BCMA), IGHG1, and IGHG4 in B cells. **e** Distribution of different subclasses of B cells in stage I and stage III NSCLC. **f** Heatmap showing the difference of BC1 (naïve-like B cells) between NSCLC and PBMC

To illustrate the characteristics of B cells in NSCLC, the cells in C4 and C6 clustered in our NSCLC scRNA-seq were kept for further analysis. We also extracted normal single-cell datasets from peripheral blood mononuclear cells (PBMCs) generated by 10X genomics. We then combined single-cell RNA-seq data of B cells in NSCLC and in the blood (Fig. 2a). The B cells could be divided into two major groups (BC1 and BC2); the PBMCs contain 91.8% BC1 cells and 8.2% BC2 cells, while the NSCLC samples contain 61.8% BC1 cells and 39.2% BC2 cells (Fig. 2b). The BC1 cells express markers of naïve B cells, such as MS4A1 (CD20), CD19, CD22, TCL1A, and CD83, and the BC2 cells express markers of the plasma B cells, such as CD38, TNFRSF17 (BCMA), and IGHG1/IGHG4 (Fig. 2c, d). To further investigate the distribution of the two B cell subtypes across different stages of NSCLC, we analyzed the percentage of the two cell types in stage I and stage III NSCLC. We found that the naïve-like B cells were significantly lower in stage III compared to stage I (*P* < 0.001 by *t* test) (Fig. 2e).

We further examined the differentially expressed genes between the BC1 (naïve-like B) cells in PBMC and the TME of NSCLC. A total of 73 differentially expressed genes were identified by using ROC analysis (Fig. 2f). The BC1 cells in NSCLC overly expressed RACK1, JUND, CD83, ELOB, NFKB1A, APOE, and GADD45B, while the BC1 cells in PBMC overly expressed GNB2L1, GLTSCR2, NBEAL1, ALDOA, and TCEB2 (Fig. 2f). We next performed cell-cell interaction analysis using the CellPhoneDB algorithm [28] and identified a cell-cell interaction network among the two cell types (and other cell types). It is noteworthy that the genes expressed by the B cells showed a strong interaction with other immune cell types (Additional file 1: Figure S2), suggesting an essential role of B cells in the microenvironment of NSCLC.

### Naïve-like B cells are associated with good prognosis of NSCLC

**Fig. 3:**
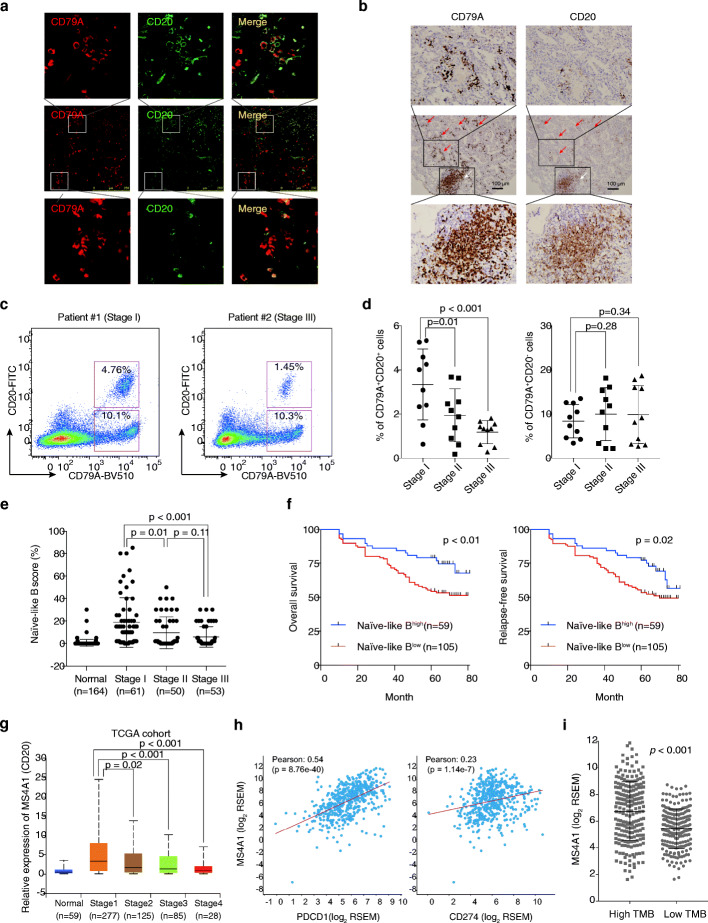
CD20^+^ B cells are associated with good prognosis of NSCLC. **a**, **b** Validation of the presence of CD79A^+^CD20^+^ and CD79A^+^CD20^−^ B cells in NSCLC tumors. Antibodies against CD79A and CD20 were used for immunofluorescence and immunohistochemistry. **a** Co-staining of CD79A (red) and CD20 (green), as well as the staining of CD79A only, was illustrated by immunofluorescence. **b** Immunohistochemistry showing the distribution of CD79A and CD20 in NSCLC tissue. The CD20^+^ B cells were located at the tertiary lymphoid structures (TLS) only, but the CD79^+^ B cells were located at both TLS and the tumor regions. **c**, **d** Flow cytometry showing the CD79A^+^CD20^+^ cells were reduced in advanced stages of NSCLC. **c** Representative illustration of flow cytometry results was plotted. **d** The ratio of CD79A^+^CD20^+^ and CD79A^+^CD20^−^ in 30 tumor tissues from I–III stages of NSCLC were plotted. **e**, **f** Patient with high levels of infiltration of naïve-like B cells was associated with better clinical outcome of NSCLC. **e** The naïve-like B score determined by immunohistochemistry in I–III stages of NSCLC was plotted. **f** The enrichment of naïve-like B cells (Naïve-like B^high^) was correlated with good prognosis of NSCLC in 164 patients. The difference in overall survival and relapse-free-survival between Naïve-like B^high^ patients and Naïve-like B^low^ patients was determined by log-rank test. The NSCLC tumors were divided into two subgroups, including Naïve-like B^high^ and Naïve-like B^low^ according to the immunostaining of CD79A and CD20 across 164 NSCLC tissues. **g** Validation using TCGA datasets. The mRNA level of MS4A1(CD20) in different stages of NSCLC as well as normal control was plotted. MS4A1(CD20) was highly expressed in lung cancer as compared to adjacent normal tissues but decreased in advanced stages of lung cancers in the TCGA cohort. **h**, **i** Correlation between MS4A1(CD20) expression and PDCD1 (PD-1)/CD274 (PD-L1) as well as tumor mutation burden of LUAD. The expression of MS4A1 (CD20) and PDCD1/CD274 was obtained from the TCGA-LUAD cohorts. The tumor mutation burden (TMB) was calculated and divided into the high TMB and low TMB groups

To validate the co-existence of different B cells in the microenvironment of NSCLC and determine the clinical relevance of each subtype of B cells, we performed co-staining of CD79A and CD20 in the primary NSCLC tissues by immunohistochemistry and immunofluorescence. As shown in Fig. 3a, a subset of cells express both CD79A and CD20, while the rest express the CD79A only. We noticed that the CD20-positive cells were mainly located at the tertiary lymphoid structures (TLS) of lung tumor tissues while CD79A-positive cells were not only located at the TLS but also randomly enriched within tumor tissues (Fig. 3b), suggesting CD20^+^ naïve-like B cells and plasma-like B cells might function versatile in the lung cancers. We further applied a flow cytometry analysis in a cohort of 30 NSCLC tumor tissues, including 10 stage I, 10 stage II, and 10 stage III fresh tissues. The flow cytometry also demonstrated the co-existence of the two subtypes of B cells (Fig. 3c). In addition, we observed a decrease of the CD20^+^CD79^+^ B cell in the advanced stages of NSCLC (Fig. 3d).

To confirm this observation, we analyzed the clinical impact of infiltrating naïve-like B cells in a cohort containing 164 NSCLC samples. We stained the 164 NSCLC samples with the antibodies against CD20 and CD79A and evaluated the infiltration level of naïve-like B cells by using the ratio of CD20^+^CD79A^+^-positive cells in TILs. We found that the infiltration level of naïve-like B cells was significantly decreased in stage II (*P* = 0.01 by *t* test) and stage III NSCLC (*P* < 0.001 by *t* test) compared to stage I NSCLC (Fig. 3e). We further divided the NSCLC samples into two groups, the naïve-like B^high^ group and the naïve-like B^low^ group, according to the infiltration level of the CD20^+^CD79A^+^ cells in tumor tissues. We then compared the prognosis of the two groups. We found that the higher infiltration levels of naïve-like B correlated with a better overall survival (*P* < 0.01 by log-rank test) and relapse-free survival (*P* = 0.02 by log-rank test) (Fig. 3f, Table 1).

**Table 1:** Clinical relevance of naïve-like B cells in NSCLC

Clinical characteristics	Naïve-like B^**low**^(***N*** = 105)	Naïve-like B^**high**^(***N*** = 59)	***P*** value
**Age**			0.129
≤ 58	44 (50.5)	32 (67.8)	
>58	61 (49.5)	27 (32.2)	
**Sex**			0.517
Male	50 (47.6)	25 (42.4)	
Female	55 (52.4)	34 (57.6)	
**Location**			0.216
Left	31 (29.5)	23 (39)	
Right	74 (70.5)	36 (61)	
**Tumor size**			0.744
≤ 3 cm	74 (70.5)	43 (72.9)	
>3 cm	31 (29.5)	16 (27.1)	
**N stage**			< 0.001
0	52 (49.5)	46 (78.0)	
1–2	53 (50.5)	13 (12.0)	
**AJCC stage**			0.006
I	30 (28.6)	31 (52.5)	
II	34 (32.4)	16 (27.1)	
II	41 (39.0)	12 (20.3)	

We further examined the expression level of subtype-specific marker genes of the two B cell types in different stages of lung cancer using the transcriptome datasets from the LUAD TCGA cohorts. We identified that the expression of MS4A1 (CD20) (Fig. 3g) were higher in tumor tissues compared to adjacent normal tissues and the expression level of MS4A1 was significantly decreased in the advanced stages of lung cancers (Fig. 3g). As the outcome of PD-1/PD-L1 inhibitors was mostly dependent on the expression level of PD-1/PD-L1 and the tumor mutation burden (TMB), we examined the association of B cell infiltration with the PD-1/PD-L1 expression and TMB in the TCGA cohort. The higher level of B cell infiltration was positively correlated with higher expression of both PD-1/PD-L1 and the TMB level (Fig. 3h, i), implying the high levels of B cell infiltration might also benefit the anti-PD-1/PD-L1-based immunotherapy.

### Naïve-like B cells inhibit cell proliferation of NSCLC

**Fig. 4:**
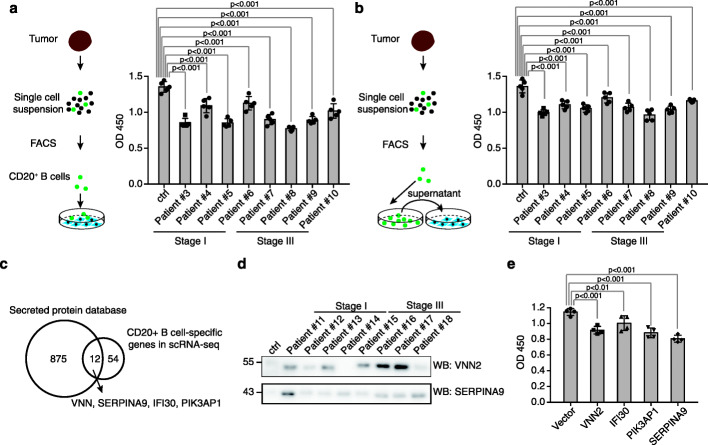
Naïve-like B cells inhibit the cell growth in lung cancer. **a** Naïve-like B cells inhibit cell growth of A549 cells. The left panel showed the experimental design. The cell growth of A549 cells co-cultured with or without CD20^+^ B cells was determined by CCK8 assays (right panel). **b** Naïve-like B cells inhibit cell growth of A549 cells in a cell-cell interaction-independent manner. The left panel showed the experimental design. The cell growth of A549 cells treated with or without culture supernatants of CD20^+^ B cells was determined by CCK8 assays (right panel). **c** t-SNE plots showing the expression and distribution of SERPINA9, VNN2, IFI30, and PIK3AP1 in single-cell RNA-seq analysis of lung tumor. **d** Determination of VNN2 and SERPINA in the culture media of naïve-like B cells from patients’ tissues. B cells isolated from PBMC were used as control (ctrl). **e** Overexpression of VNN2, IFI30, PIK3AP1, and SERPINA9 inhibited the cell growth of A549 cells

To assess the direct effects of the naïve-like B cells on tumor cells, we applied the flow cytometry sorting using antibodies against the CD20 to obtain the naïve-like B cells in NSCLC tissues. The naïve-like B cells in 8 fresh NSCLC tumor tissues, including 4 stage I and 4 stage III tumor tissues, were sorted. We then co-cultured the naïve-like B cells with lung cancer cell lines A549 and H1299 in vitro. Cell viability of the A549 and H1299 cells after the co-culture with CD20^+^ B cells was examined at 24 h. The co-culture with CD20^+^ B cells significantly inhibited the growth of A549 and H1299 cells (*P* < 0.001) (Fig. 4a, Additional file 1: Figure S3a).

Since the effects of immune cells on malignant cells could be achieved either by a direct cell-cell interaction or via soluble mediators such as factors and cytokines, we extracted the culture supernatant of the CD20^+^ B cells isolated from the NSCLC tissue and then used it to treat the A549 and H1299 cells for 24 h. We found the culture supernatant of the CD20^+^ B cells also suppressed the growth of A549 and H1299 cells (Fig. 4b, Additional file 1: Figure S3b), indicating the effects of the CD20^+^ B cells on lung cancer cells can be independent of cell-cell interaction and also suggesting that the CD20^+^ B cell-secreted proteins might contribute to the tumor-suppressive function of CD20^+^ B cells in NSCLC.

To further determine which factors secreted by B cells were involved in the regulation of tumor cells, we firstly analyzed the genes abundantly expressed in the CD20^+^ B cells. 1426 highly expressed genes in CD20^+^ B cells were identified, and 66 uniquely expressed genes were identified (Fig. 4c). We then acquired the human secreted protein in the Uniprot database and overlapped the highly expressed genes in the CD20^+^ B cells with the annotated secreted proteins. Four secreted proteins were uniquely expressed in the CD20^+^ B cells, including VNN2 (Vanin 2), IFI30 (Gamma-Interferon-Inducible Protein IP-30), PIK3AP1 (B Cell Adaptor Protein), and SERPINA9 (Serpin Family A Member 9) (Fig. 4c). Next, to confirm the secretion of SERPINA9 and VNN2 from CD20^+^ B cells, we determined the protein level of SERPINA9 and VNN2 in the culture media of B cells. We found that SERPINA9 and VNN2 were enriched (Fig. 4d), indicating the secretion of these proteins by CD20^+^ B cells in NSCLC.

Next, we tried to determine the effects of these four proteins on tumor cells. However, due to the limited commercialized protein of these four proteins, we were unable to check the direct effects of these four proteins on tumor cells. We thus overexpressed these genes in A549 and H1299 lung cancer cell lines to test the effects of these proteins on NSCLC cells. We found that the overexpression of SERPINA9 and VNN2 inhibited the growth of A549 and H1299 cells (Fig. 4e, Additional file 1: Figure S3c).

### Plasma-like B cells in the advanced-stage NSCLC promote tumor cell proliferation while those in the early-stage NSCLC partially inhibit the proliferation

**Fig. 5:**
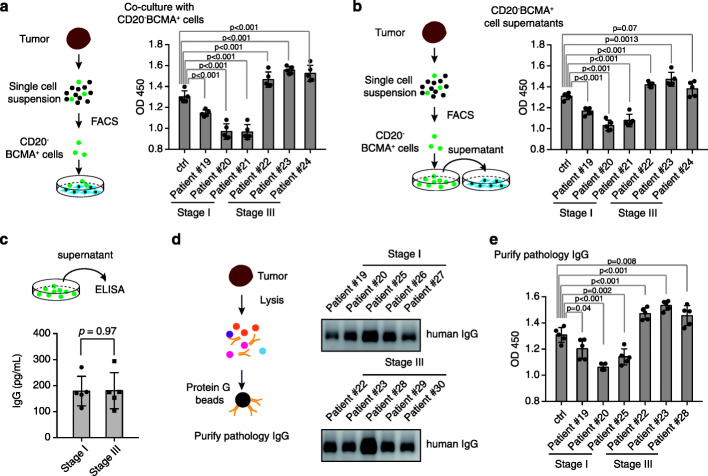
IgG-producing plasma-like B cells exert a different effect on tumor cells in different stages of lung cancer. **a** IgG-producing plasma-like B cells isolated from patient at stage III lung cancers promote proliferation of A549 cells. The left panel shows a schematic of the experiment design. CD20^−^BCMA^+^ B cells isolated from stage I or stage III lung tumors were co-cultured with A549 cells, and the cell growth was determined by CCK8 assays (right panel). **b** The function of CD20^−^BCMA^+^ B cells on A549 cells was exerted in a cell-cell interaction-independent manner. The left panel showed a schematic of the experiment design. The culture supernatant of the CD20^−^BCMA ^+^ B cells isolated from stage I or stage III lung tumors was used to treat A549 cells, and the cell growth was determined by CCK8 assays (right panel). **c** The level of IgG produced by plasma-like B cells had no significant difference between stage I and stage III. The amount of IgG was determined by ELISA experiments. **d** The pathology IgG in stage I and stage III exerts different functions on A549 cells. The left panel showed a schematic of the experiment design. The purified IgG was validated by western blot using antibodies against the human IgG (right panel). **e** The pathology IgG isolated from stage III NSCLC but not stage I promoted the cell growth of A549 cells

To further identify the effects of the plasma-like B cells on the tumor progression of NSCLC, we sorted the plasma-like B cells from three stage I tumor tissues and three stage III tumor tissues using antibodies against BCMA, a marker identified to be uniquely expressed in the plasma-like B cells in our scRNA-seq data. We then co-cultured the plasma-like B cells with A549 and H1299 cell lines. Interestingly, we found that the plasma-like B cells from stage III promote proliferation while the plasma-like B cells from stage I inhibit proliferation of A549 and H1299 cells (Fig. 5a, Additional file 1: Figure S4a), implying pro-tumor effects of plasma-like B cells in stage III NSCLC and a tumor-suppressive effects of the plasma-like B cells in stage I NSCLC .

To test whether the effects exerted by these plasma-like B cells relied on a direct cell-cell interaction or through the secreted molecules, we collected the culture supernatants of plasma-like B cells isolated from stage I or stage III NSCLC and then treated the A549 and H1299 cells. We also found that the culture supernatants of plasma-like B cells isolated from stage I inhibited the cell growth of A549 and H1299 cells, while the culture supernatants of plasma-like B cells isolated from stage III promoted the cell growth of A549 and H1299 cells (Fig. 5b, Additional file 1: Figure S4b), indicating that the effects exerted by the plasma-like B cell also can be attributed to the secreted molecules.

As the scRNA-seq demonstrated a high level expression of IGHG1, IGHG4 gene (encoding the IgG proteins) in the plasma-like B cells (Fig. 2d). We next determined the IgG and IgA expression of the culture media of these plasma-like B cells and observed a very high level of IgG (Fig. 5c). To ascertain the function of IgG, we isolated the IgG from different stages of NSCLC (Fig. 5d) and treated the A549 with these IgG. We found that the IgG isolated from stage I NSCLC exerts minor toxic effects on A549 cells while the IgG isolated from stage III NSCLC significantly accelerated the growth of A549 cells (Fig. 5e). Together, these observations suggested that the effect of plasma-like B cells in different stages of NSCLC might function differently on tumor cells, and the effects were mainly attributed to the secreted IgGs.

### Identification of the targets of pathology immunoglobulins

**Fig. 6:**
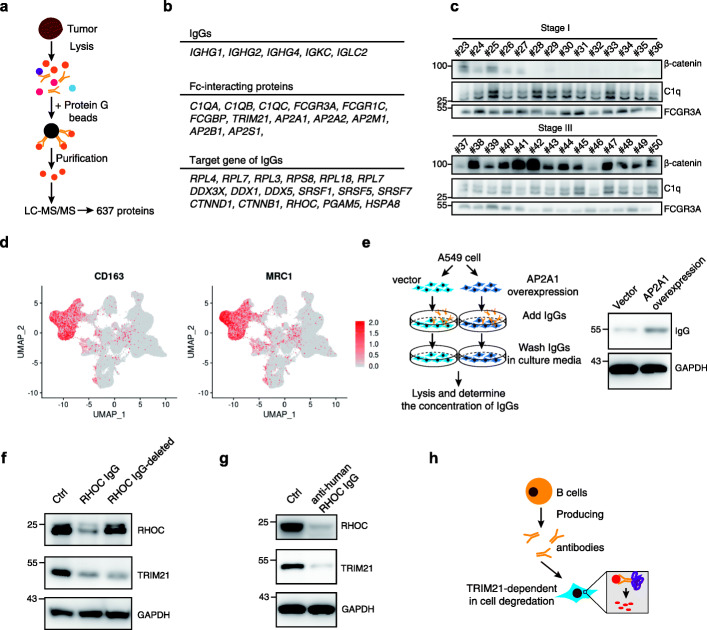
Identification of the pathologic targets. **a** Schematic illustration of the identification of the targets of pathologic antibodies. **b** Representative illustration of the proteins that interacted with pathologic antibodies. **c** Validation of the proteins bound by the pathologic antibodies. **d** The macrophages in the microenvironment of the NSCLC mainly belong to the M2 macrophages. The expression of M2 macrophage-specific marker CD163 and MRC1 (CD206) in the single-cell dataset was plotted. **e** Overexpression of AP2A1 results in increased endocytosis of IgGs. Left panel shows the experimental design. A549 cells were transduced with AP2A1 overexpression or vector plasmid and treated with IgG. Cellular IgGs were determined by western blot (right panel). **f** A549 cell lines were electroporated with PBS (ctrl), pathology RHOC IgGs, and RHOC-IgG depleted IgGs, and the whole cell lysates were harvested 3 h after electroporation. The expression levels of RHOC and TRIM21 were determined by western blot. GAPDH was used as the internal control. **g** A549 cell lines were electroporated with PBS (ctrl) or recombinant rabbit antibodies against RHOC, and the whole cell lysates were harvested 3 h after electroporation. The expression levels of RHOC and TRIM21 were determined by western blot. GAPDH was used as the internal control. **h** Schematic of TRIM21-mediated degradation of specific targets in tumor cells

To illustrate the function of these IgGs produced by plasma-like B cells, we further identified the targets of the IgGs in cancer cells. We performed an immunoprecipitation using the endogenous IgG produced by the plasma-like B cells in 8 NSCLC tissues, including 4 stage I and 4 stage III NSCLC. We firstly performed cell lysis on tumor samples, which contains the IgGs and the target proteins of these IgGs as well, and precipitated these IgGs as well as their targets using magnetic protein A/G beads. The precipitated proteins were then subjected to for proteomics analysis (Fig. 6a). A total of 637 proteins were identified (Additional file 4: Table S3).

These proteins could be classified into three groups: the IgG protein, the IgG-binding proteins, and the targets of the IgGs (Fig. 6b). The LC-MS/MS was further validated by western blot (Fig. 6c). The IgG proteins are directly captured by the protein A/G beads, including the IGHG and IGHL. The IgG-binding proteins are proteins which directly interacted with IgG, mainly the Fc-binding proteins. These proteins bind to the IgG through the Fc-domain of IgG and trigger the downstream biological events. We next analyzed the expression of these Fc-binding proteins across different cell types and found that the Fc-binding proteins were mainly expressed by monocytes or macrophages (Additional file 1: Figure S5), such as C1QA, C1QB, C1QC, FCGR3A, and FCGR1C, which might contribute to the antibody-dependent cellular cytotoxicity.

We checked the characteristic of the macrophages infiltrated in the microenvironment of NSCLC. The macrophages in NSCLC tissues express high levels of M2 macrophage markers, including CD163 and CD206 (MRC1) (Fig. 6d), which functions by promoting the tumorigenesis other than anti-tumorigenesis. Since the antibody-dependent cell-mediated cytotoxicity (ADCC) mainly attributed by the M1 macrophages, these antibodies were most likely not be able to initiate the ADCC in the microenvironment of NSCLC, despite a potential interaction with the macrophages.

We next explored the ADCC-independent function of these antibodies. Many new targets of IgGs in lung cancer were non-cell surface proteins, but we noticed that many targets of these IgGs were adaptor proteins, such as the AP-2 complex including AP2A1, AP2A2, AP2B1, AP2S1, and AP2M1(Fig. 6b). As the AP-2 complex was mainly reported to be involved in endocytosis, it is tempting to speculate that some IgGs might be endocytosed to other cells via the AP-2 complexes. To test this hypothesis, we first determined the expression of AP-2 in the microenvironment of lung cancers. The AP-2 protein is highly expressed in the tumor cells as well as the monocytes in lung cancer (Additional file 1: Figure S5). Next, to assess the role of AP-2 in the transportation of IgGs, we applied a cellular model for IgG determination. We overexpressed the AP2A1, a core component of AP2 complex that was highly expressed in tumor tissue of lung cancers, in A549 cells and added purified human IgGs to cell culturing media. The IgGs were significantly increased in cells with AP2A1 overexpression (Fig. 6e), indicating the IgGs would be delivered into tumor cells in an AP2-dependent manner.

Next, to address the potential functions of the IgGs transported into tumor cells, we reviewed the IgG-binding proteins identified in the aforementioned protein-omics and cellular function of IgGs. We noticed that TRIM21, an Fc-binding protein involved in ubiquitin-dependent antibodies-targets degradation [29], was significantly enriched in the targets of pathology IgGs (Fig. 6b, Additional file 4: Table S3). Thus, it is tempting to speculate that the pathology IgGs might contribute to the degradation of specific targets via its association of TRIM21 in tumor cells. To address this possibility, we choose Rhoc as one of the representative cellular targets of IgGs identified in LC-MS/MS together with the expression of these targets in tumor cells using the scRNA-seq results (Fig. 6b, Additional file 1: Figure S5, Additional file 4: Table S3). Rhoc was reported to be an oncogene that was overexpressed in NSCLC cells and involved in tumor cell proliferation and metastasis.

We electroporated the pathology Rhoc IgGs and the Rhoc-depleted IgGs into A549 and H1299 cells, respectively. We found that the expression of Rhoc was significantly decreased upon Rhoc-IgG treatment, but the inhibition was decreased upon deletion of Rhoc-IgG (Fig. 6f). Furthermore, we electroporated a monoclonal rabbit anti-human Rhoc IgGs into A549 cells and observed similar results as the pathology Rhoc-IgG (Fig. 6g), suggesting the pathology Rhoc-IgG might trigger the TRIM21 pathway and lead to degradation of the Rhoc proteins. Together, these results suggest a novel role of IgGs on tumor cells, i.e., the IgGs could be delivered into the tumor cells with the help of AP2 complex. The imported IgGs recognized their targets through the Fab-domains while the Fc-domains interact with TRIM21 and triggered the ubiquitin-mediated degradation of RHOC in tumor cells (Fig. 6h).

## Discussion

Immune checkpoint inhibitor has achieved significant success in treating melanoma and blood cancers, but its efficacy on solid cancer types remains relatively low [3, 30]. B cell is one major class of immune cells in the adaptive immune systems and has multiple functions in facilitating the co-evolution of cancer and its microenvironment. However, the functions of tumor-infiltrating B cells remain incompletely characterized. Here, we identified novel subtypes of B cells infiltrated in NSCLC via a single-cell RNA-seq experiment, and revealed multiple novel functions of subtypes of tumor-infiltrated B cells in NSCLC, including (I) the infiltrated B cells could be divided into two classes with distinct gene expression signatures, (II) CD20^+^ B cells inhibit tumor growth in NSCLC, (III) the IgG^high^ B cells produce immunoglobulin and inhibit cell growth in the early stage of NSCLC but may promote cell growth in the advanced stage of NSCLC, (IV) proteomics profiling identified the novel targets and functions of the pathological antibodies produced by plasma-like B cells, and (V) the pathological antibody could be imported into tumor cells via AP2 complexes and degrade its targets through the TRIM21-mediated ubiquitin pathway.

Although CD20 and CD19 are the most prevalently used markers of B cells, we identified a subset of CD20^−^CD19^−^CD79A^+^CD79B^+^ B cells in NSCLC. Comparing to CD19/CD20-positive B cells, CD79A/CD79B are more generally expressed in the B cells through different maturation and function stages [31]. We identified that the CD20^+^ B cells were mainly located in TLS. TLS density is positively correlated with patient’s favorable clinical outcome in lung cancer. Recent studies also suggested that TLS formation is associated with cancer patients’ response to immunotherapy [32–35]. Our results revealed that the CD20^+^ B cells may inhibit the growth and progression of tumor cells in the early disease stage, and positively correlated with good prognosis of NSCLC. We also identified overly expressed secreted proteins, such as VNN2, IFI30, PIK3AP1, and SERPINA9, in the CD20^+^ B cells. Current studies in tumor-associated B cells, such as the regulatory B cells, mainly focused on the secretion of IL-10 and TGF-β, which might contribute to the function of T cells and therapy effect of T cell-mediated immune response [36]. Our study revealed that the CD20^+^ B cells in the microenvironment of lung cancers produce a high level of VNN2 and SERPINA9 and directly inhibit the growth of NSCLC cells. A possible future direction is to comprehensively characterize the functions of the proteins secreted by CD20^+^ B cells.

Clinically, our studies emphasized on the prognostic impact of naïve-like B cells as well as the roles of the genes uniquely expressed by naïve-like B cells, such as TLR10, FCRL1, BLK, and TNFRSF13B in NSCLC. Previous studies have revealed infiltrated B cells are related with poor prognosis of gastric cancer [37] and prostate cancer [38], but with favorable prognosis of NSCLC and breast cancer [39]. Here, by integrating our single-cell transcriptomic data with TCGA data, we further confirmed that the CD20^+^CD79A^+^ B cells were a favorable factor in NSCLC. Our newly identified CD20^+^ B cells and their uniquely expressed genes could potentially be used to predict patients’ prognosis of NSCLC.

We demonstrated the versatile functions of B cell in NSCLC via a series of experiments, suggesting targeting a distinct subset rather than complete B cell might be a future perspective for B cell-based immune therapy. Moreover, our single-cell transcriptomics data also provides a reference map of genes that uniquely expressed in the distinct subtype of B cells in NSCLC comparing to the B cells collected from blood samples. Another future direction is to interpret the distinct function of the plasma-like specific, as well as the specifically expressed genes (such as CD38, MZB1), and their possible roles in cell-cell interactions and cancer-microenvironment co-evolution in NSCLC.

We also presented a novel mechanism for endocytosis of antibodies in the tumor cells of NSCLC. Our analysis revealed that the antibodies directly bound to the AP-2 complexes in lung tumors. The AP2 (assembly polypeptide 2) complex is one of the most abundant adaptors and a major player in clathrin-mediated endocytosis [40]. Previous studies have revealed its role in the regulation of endocytosis of integrins [41]. In this study, we found that immunoglobulin directly interacted with AP2 complex in NSCLC, and overexpressed AP2 increased the cellular immunoglobulin levels, suggesting the direct involvement of AP2 in the endocytosis of immunoglobulins in the tumor cells of NSCLC. While the function of immunoglobulin in targeting extracellular proteins has been intensively studied, the role of cellular immunoglobulin in tumor cells remains largely unknown. We demonstrated that the immunoglobulins directly interacted with TRIM21 (Tripartite Motif Containing 21). TRIM21 is an E3 ubiquitin-protein ligase and a cytosolic Fc receptor, which bound to the Fc-chains of antibody and deliver ubiquitin-proteasome to Fab-recognizing targets [29]. A recent study finds that antibody-TRIM21-ubiquitin-proteasome could be used as a tool for the degradation of specific targets [42]. We reported that the pathology Rhoc antibody could be delivered into the tumor cells and resulted in decreased Rhoc protein levels in NSCLC cell lines. Since the Rhoc mainly functions as an oncogene, the imported Rhoc antibodies might negatively regulate tumor progression, suggesting a potential anti-tumor role of these IgGs.

In summary, our studies identified multiple novel insights of B cell subtypes and immunoglobulins in regulating the progression of NSCLC. The newly established versatile function of B cells, as well as the antibodies, highlighted possible clinical implications of targeting B cell subtypes in the microenvironment of NSCLC. In addition, our single-cell transcriptome and proteomics profiling immunoglobulin-antigen provide important resources for future investigation of B cells and other immune/stromal cells in NSCLC, which may eventually benefit the clinical practice.

## Methods

### Human tumor specimens

A total of 164 paraffin-embedded samples of primary NSCLC were collected from NSCLC patients from 2012 to 2013 at the Shanghai Pulmonary Hospital. None of these patients received any anti-cancer therapy before tumor resection. The patients’ relapse-free survival (RFS) and overall survival (OS) durations were defined as the period from initial surgery to clinically proven metastasis or recurrence and death, respectively. The median patient follow-up time was 63 months after surgery (range, 5–81 months).

### Cell lines

A549 and NCI-H1299 cells were obtained from the cell bank of Chinese Academy of Science. These cells were cultured in Dulbecco’s modified Eagle’s medium (DMEM) (Gibco, Carlsbad, CA, USA) supplemented with 10% fetal bovine serum (FBS) (Gibco). All cell lines used in this study were authenticated by STR sequencing. Cells were routinely tested, and all were mycoplasma negative.

### Single-cell transcriptome profiling

The single-cell suspension of tumors was obtained by digesting with 2 mg/mL collagenase in DMEM supplemented with 10% FBS. Non-digestible tissues were filtered with the 70-μm Cell Strainer (BD Falcon). The mononuclear cell was then separated using the Ficoll-Paque PLUS density gradient media (GE health). Single-cell library was constructed with the Chromium Single Cell 3′ Reagent Kits (v2 Chemistry) according to manufacturer’s instructions. Libraries were sequenced at the illumine platforms. The data matrix of each single cells was analyzed using the Cell Ranger Pipelines.

Briefly, raw sequences were processed with Cell Ranger (version 3.0.2), a package that takes Illumina bcl or fastq files as input and generates a count matrix after filtered errors and biases. Filtered reads were aligned to the human genome (GRCh38) with STAR [43], a high-performance community-standard aligner. After alignment, reads were translated into a UMI matrix. The matrix of read counts per gene per sample was further analyzed by the Seurat suite (version 3.1.0) [44] in the R program (version 3.6.0). A total of 144,271 cells that passed quality control steps implemented in Cell Ranger were obtained. We further applied a quality control to get highly reliable cells; cells meeting any of the following criteria were excluded: < 500 or > 6000 unique genes expressed, > 40,000 UMIs, or > 50% of reads mapping to the mitochondria. These steps removed an additional 28,726 cells, resulting in a final dataset of 115,545 cells. We quantified gene expression across 35,538 genes, of which 19,540 were expressed in at least one cell.

To keep a standard procedure for clustering, we used a value of 0.5 for the resolution. To investigate transcriptional heterogeneity and to undertake initial cell clustering, we applied a dimensionality reduction with principal component analysis (PCA). We selected the top 30 principal components (PCs) that explained more variability than expected by chance using a permutation-based test in Seurat. For the cell clustering within primary clusters (subclustering), we selected variable numbers of PCs for dimensionality reduction using either permutation-based analyses or heuristic methods in Seurat. We used PC loadings as input for a graph-based approach to cluster cells by cell type and as input for uniform manifold approximation and projection (UMAP) for reduction to two dimensions for visualization purposes.

Cluster-specific genes were acquired using the FindMarkers algorithm in the Seurat suite. Test used for cell marker identification was ROC analysis. Cell types of each cluster were determined by scMatch [27] and manually checked with known cell surface markers. The PBMC scRNA-seq datasets were obtained from the database of 10X genomics (https://support.10xgenomics.com/single-cell-gene-expression/datasets). The clusters expressing CD79A or MS4A1 were identified as B cells. Cell barcodes of the B cell clusters and corresponding gene counts were extracted for generating B cell expression matrix.

### Identification of the correlation of gene expression with the survival of LUAD in TCGA cohorts

The expression of each gene and the clinical information of each patient with LUAD were obtained from the TCGA cohorts [45]. The coefficient values were obtained from OncoLnc database (http://www.oncolnc.org/download/). The top 10 genes in each cluster of the scRNA-seq data were used for plotting of the Cox coefficient values. The average of the Cox values of the 10 genes in each cluster was calculated.

### Flow cytometry

The single-cell suspension of tumors was isolated as described above. Non-digestible tissues, as well as the pellets of dead cells, were filtered with the 70-μm Cell Strainer (BD Falcon). Surface staining was performed with anti-CD79A (562,852, BD #300412) and anti-CD20 (561741, BD Biosciences #560273) antibodies. Cells were then resuspended in 400 μL PBS and analyzed on LSRII flow cytometer (BD Biosciences).

### Histology, immunohistochemistry (IHC), immunofluorescence (IF)

Serial 4-mm-thick formalin-fixed paraffin-embedded (FFPE) tissue sections were baked in a drying oven at 60 °C for 1 h. Then, they were deparaffinized in xylene and rehydrated in decreasing concentrations of ethanol. Tissue sections were incubated in retrieval solution (citrate buffer, pH 6) for antigen retrieval at 95 C for 30 min. Tissue sections were incubated in 3% hydrogen peroxide and in serum-free protein block solution (Dako, X0909) before adding the primary antibody (CD20, ab78237, abcam; CD79A, ab199001, abcam) for 1 h at room temperature. After signal amplification using biotinylated-secondary antibody and streptavidin-horseradish peroxidase, slides were counterstained with hematoxylin, mounted with a glycerol-based mounting medium, and scanned for digital imaging. The TILs were composed of mononuclear cells, including lymphocytes. Intra-alveolar macrophages were not considered as part of the immune infiltration [32]. All IHC results were independently scored by two pathologists (Prof. Likun Hou and Chunyan Wu). In case of disagreement, the slides were re-examined until the observers reached a consensus. A semiquantitative manual scoring was used to evaluate the percentage of TILs exhibiting membrane staining (positive staining proportion score). The overall infiltration of the naïve-like B cells was quantified by multiplication of the ratio of CD20^+^CD79^+^ double-positive cells among the TILs cells. We defined the ratio > 10% as naïve-like B^high^ cells, while the ratio ≤ 10% as naïve-like B^low^ cells. Meanwhile, immunofluorescence was performed using anti-CD79A (562852, BD) and anti-CD20 (555622, BD) antibodies. Immunofluorescent images were recorded with a LeicaTCS-SP5 confocal laser scanning microscope (Heidelberg, Germany). All immunostained slides and matching hematoxylin and eosin-stained sections were scanned with the MoticEasyScan digital scanner.

### Western blot analysis

Total protein was isolated using RIPA solution (Beyotime Biotechnology, Jiangsu, China), and BCA protein assay kit (Beyotime Biotechnology, Jiangsu, China) was applied to detect the concentration of the protein. Equal amounts of the protein (50 μg) were spotted into 10% SDS-PAGE gel for electrophoresis, followed transferred onto PVDF membranes. Then, the membranes were soaked into 5% milk for an hour at room temperature. Subsequently, membranes were incubated at 4 °C overnight with primary antibodies against primary antibody, After that, the membranes were washed by PBS and incubated with HRP-tagged secondary antibody for 1 h. The proteins were then detected using ECL reagent (Pierce Biotechnology, USA). GAPDH levels served as loading controls. All experiments were repeated in triplicate. Primary antibodies were as follows: anti-human AP2A1 (ab61, Abcam), anti-human RHOC (#3430, Cell Signal Technology), and anti-human TRIM21 (ab207728, Abcam).

### B cell isolation and stimulation

The mononuclear cells among tumors were separated using the Ficoll-Paque PLUS density gradient media (GE health). The pellets of dead cells were filtered with the 40-μm Cell Strainer (BD Falcon). And the mononuclear cells were stained with anti-BCMA (357503, Biolegend) or anti-CD20 (555622, BD Biosciences) antibodies. BCMA-positive or CD20-positive cells were sorted with the BD FACSAria II. The sorted cells were cultured in RPMI 1640 Medium supplemented with 10% FBS and 200 U/mL penicillin-streptomycin. After being sorted for 24 h, cells were centrifuged and resuspended in fresh RPMI 1640 Medium supplemented with 10% FBS. The supernatant of sorted cells was collected 48 h after culture. For co-culture experiments, the sorted cells were co-cultured with A549 and H1299 cells in DMEM supplemented with 10% FBS for 48 h. After 48 h of co-culture, B cell-containing culture media were removed and replaced with fresh culture media and cultured for further 4 h, and the cell viability of A549 and H1299 cells was determined by CCK8 assays.

### Purification of immunoglobulin by affinity chromatography

The tumors were lysed in dounce homogenizer with IP buffer (20 mM Tris-HCL (PH 8.0), 2 mM EDTA, 1% Triton-X100, 150 mM NaCl, 1× proteinase inhibitor cocktail). The supernatants were collected and subjected to IgG-target isolation. Maganic Protein G beads were added to the tumor lysate and incubated at 4 °C overnight. For western blotting and LC-MS/MS, the beads were washed with IP buffer for 5 times and boiled in 2× SDS loading buffer. Purification of the pathology anti-RHOC was performed as described. Briefly, the GST-RHOC was immobilized with GSTrap HP columns. After loading with IgG isolated from tumors, the column was washed twice with binding buffer. The non-bound immunoglobulin and elutions were collected.

### Statistical analysis

GraphPad Prism 7.0a was used for statistical analysis with the RT-qPCR results, IHC quantification, flow cytometry analysis, OD45 data, and survival analysis. *P* values from unpaired two-tailed Student’s *t* tests were used for estimating the statistical significance between different groups. *P* values from the log-rank test were used for survival analysis.

## Supplementary information

**Additional file 1: Figure S1.** Representative illustration of gene expression in single cell RNA-seq. **Figure S2.** Identification of cell-cell interactions between different cells in the microenvironment of NSCLC. **Figure S3.** CD20^+^ B cells inhibit the cell growth and invasion in H1299 cells. **Figure S4.** Plasma-like B cells exert a different effect on tumor cells in different stages of lung cancer. **Figure S5.** Representative illustration of TRIM21, FCGR3A, C1QA, C1QB, C1QC, AP2A1, AP2M1, AP2A2, RHOC, CTTND1 and CTNNB1 expression.

**Additional file 2: Table S1.** Cell cluster specific gene identified by serut.

**Additional file 3: Table S2.** Prognosis of gene expression in NSCLC using the TCGA cohorts.

**Additional file 4: Table S3.** Targets of pathology antibodies in NSCLC tumors.

**Additional file 5.** Review history.

## Data Availability

The PBMC scRNA-seq datasets were obtained from the official website of 10X genomics (https://support.10xgenomics.com/single-cell-gene-expression/datasets) [46]. The complete raw data (fastqs) of the 11 scRNA-seq datasets are available at the Sequence Read Archive (SRA) database under PRJNA634159 (https://www.ncbi.nlm.nih.gov/sra/PRJNA634159) [47]. Raw data for the proteomic analysis in Fig. 6b are listed in Table S3. All materials described in this study are freely available upon request.
