# Supplementary material for: Single-cell transcriptome and antigen-immunoglobin analysis reveals the diversity of B cells in non-small cell lung cancer
Source: Genome Biol. 2020 Jun 24;21:152. doi: 10.1186/s13059-020-02064-6 (PMC7315523; doi:10.1186/s13059-020-02064-6)
Supplement: Supplementary file 1 — Additional file 1: Figure S1. Representative illustration of gene expression in single cell RNA-seq. Figure S2. Identification of cell-cell interactions between different cells in the microenvironment of NSCLC. Figure S3. CD20+ B cells inhibit the cell growth and invasion in H1299 cells. Figure S4. Plasma-like B cells exert a different effect on tumor cells in different stages of lung cancer. Figure S5. Representative illustration of TRIM21, FCGR3A, C1QA, C1QB, C1QC, AP2A1, AP2M1, AP2A2, RHOC, CTTND1 and CTNNB1 expression. [file 13059_2020_2064_MOESM1_ESM.pdf]

Supplementary figures

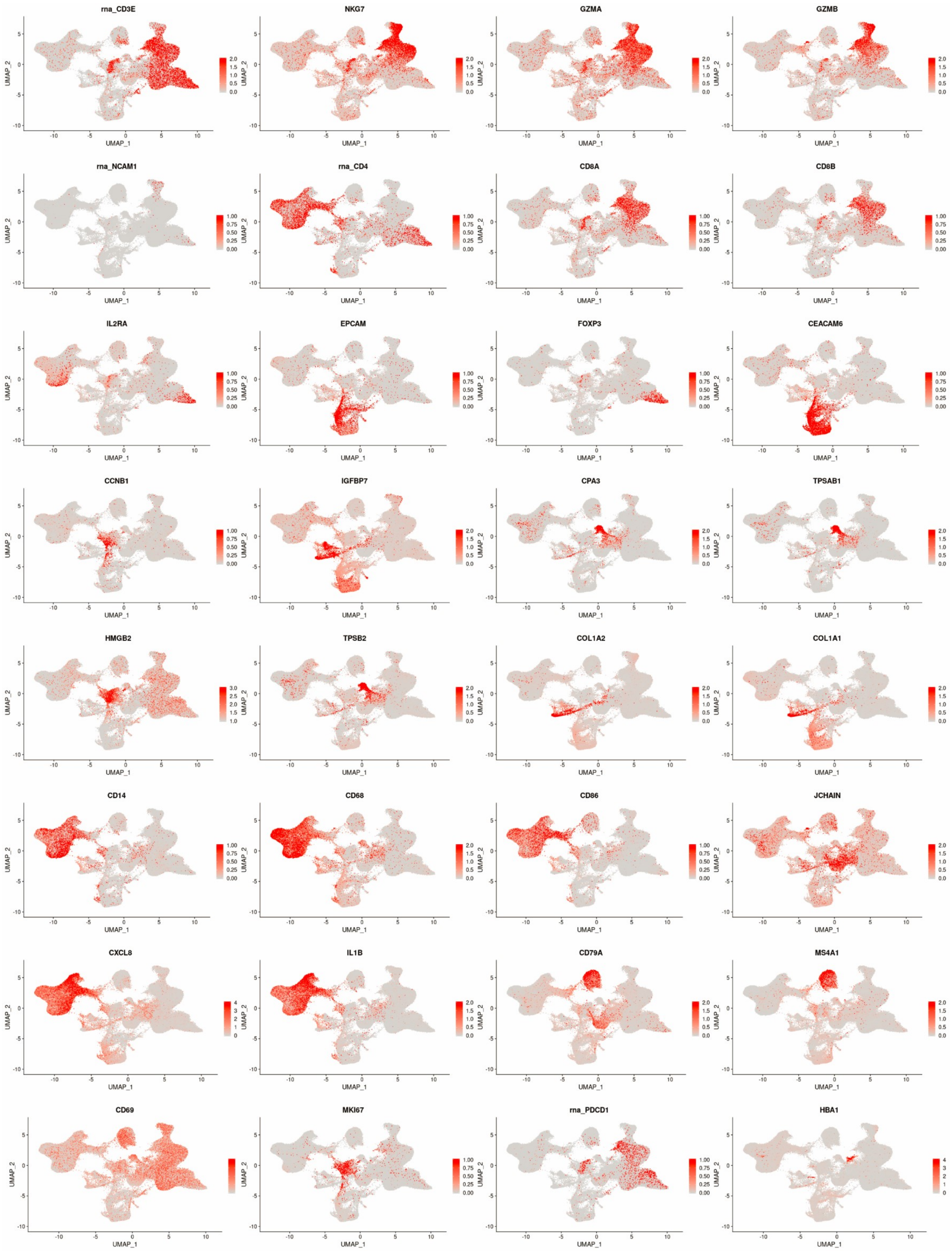

Fig. S1 Representative illustration of gene expression in single cell RNA-seq. The expression of the

representative cell type-specific genes among the 115,545 cells was plotted in the UMAP clustered graph. The scale bars were plotted in the right.

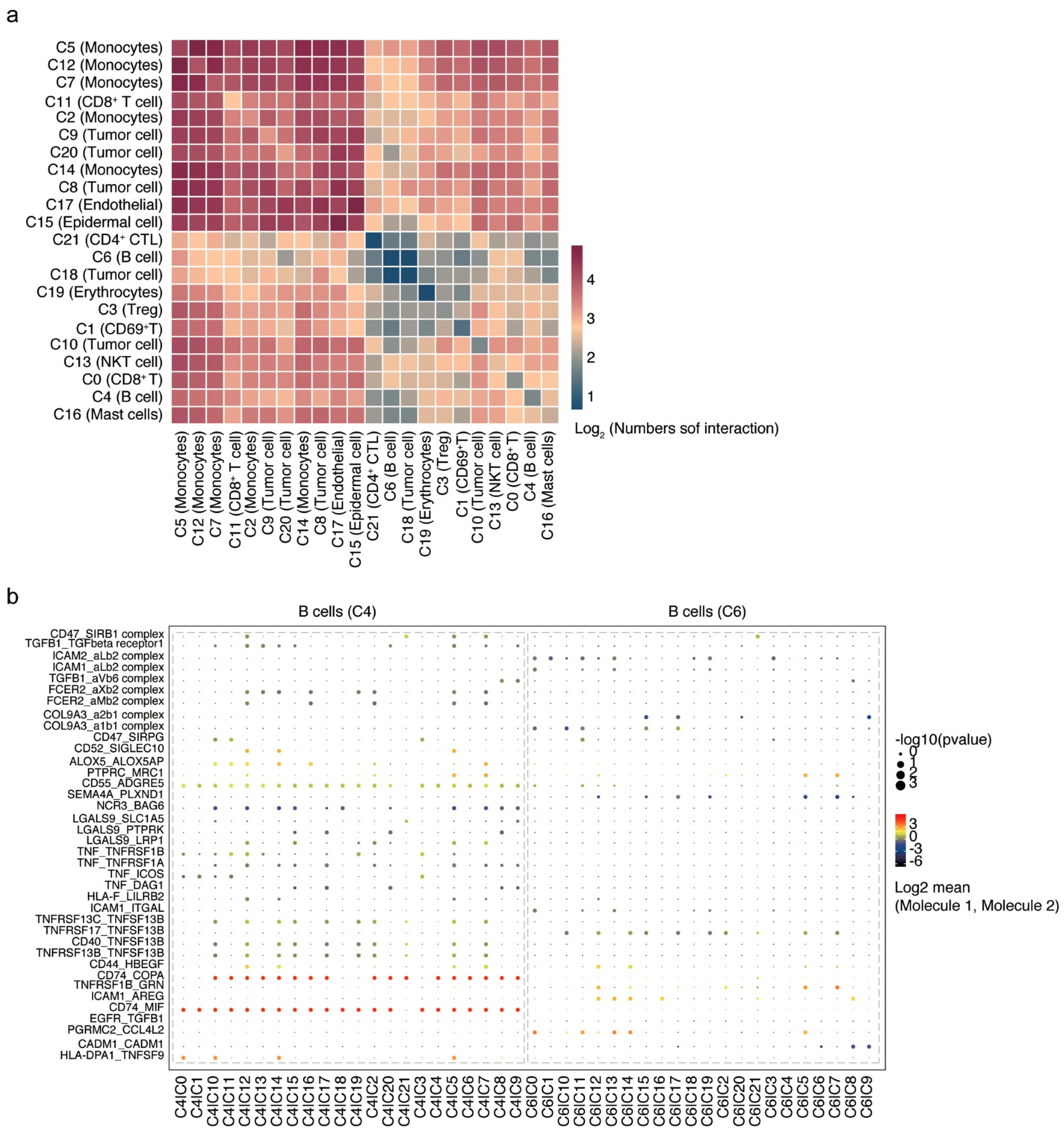

Fig. S2 Identification of cell-cell interactions between different cells in the microenvironment of NSCLC. **a** Heatmap showing the predicted cell-cell interaction. Red represents high levels of interaction and the blue represents low levels of interaction. The cell-cell interaction was determined by the expression of ligand-receptor pair genes in scRNA-seq data with the CellPhoneDB software. **b** Dot plot showing the selected

expression paired receptor-ligand genes in B cells and other cells. The average expressions of interacting genes are indicated by color. The size of the circle represents -log 10 (p value).

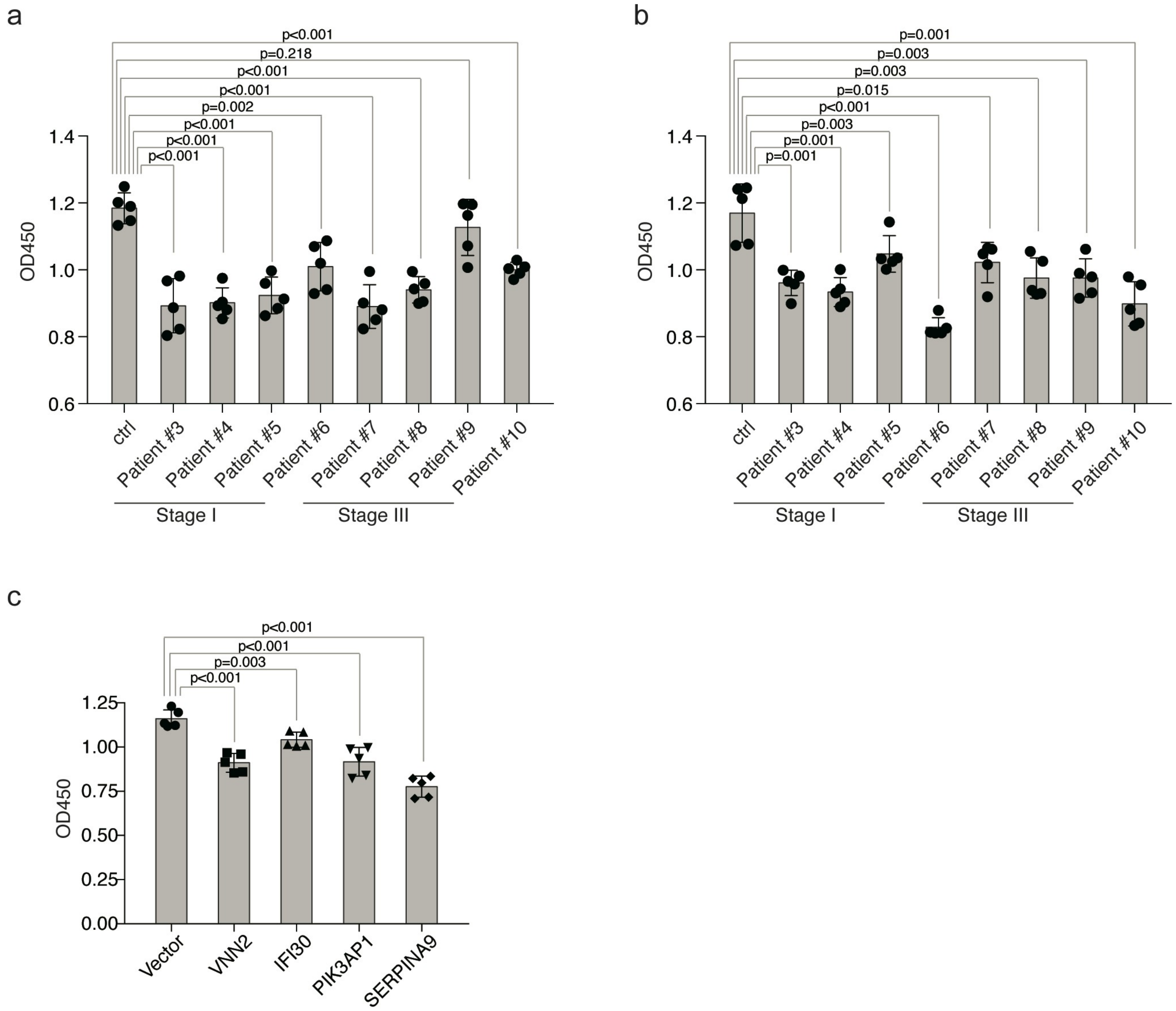

Fig. S3 CD20$^+$ B cells inhibit the cell growth and invasion in H1299 cells. **a** CD20$^+$ B cells inhibit cell growth of H1299 cells. The cell growth of H1299 cells that co-cultured with or without CD20$^+$ B cells was determined by CCK8 assays. **b** CD20$^+$ B cells inhibit cell growth of H1299 cells in a cell-cell-interaction independent manner. The cell growth of H1299 cells treated with or without culture supernatants of CD20$^+$ B cells were determined by CCK8 assays. **c** Overexpression of VNN2, IFI30, PIK3AP1 and SERPINA9 inhibited cell growth of H1299 cells.

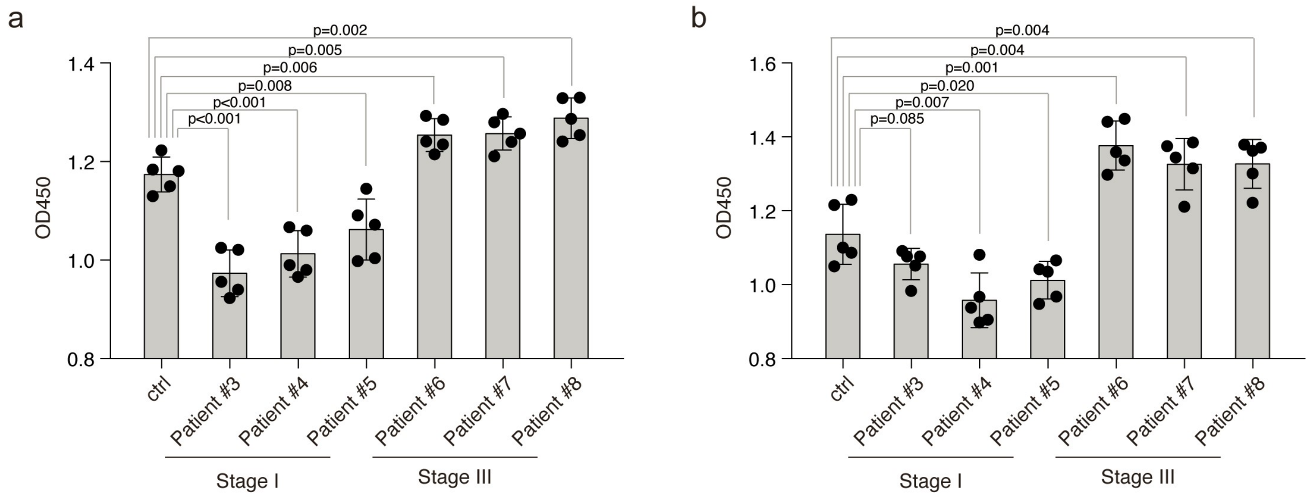

Fig. S4 Plasma-like B cells exert a different effect on tumor cells in different stages of lung cancer. **a** Plasma-like B cells isolated from stage III NSCLC promote proliferation of H1299 cells. BCMA$^+$ B cells isolated from stage I or stage III NSCLC were co-cultured with H1299 cells, and the cell growth was determined by CCK-8 assays. **b** Plasma-like B cells regulated H1299 cells in a cell-cell-interaction independent manner. The culture supernatant of the BCMA$^+$ B cells isolated from stage I or stage III NSCLC was used to treat H1299 cells, and the cell growth was determined by CCK-8 assays.

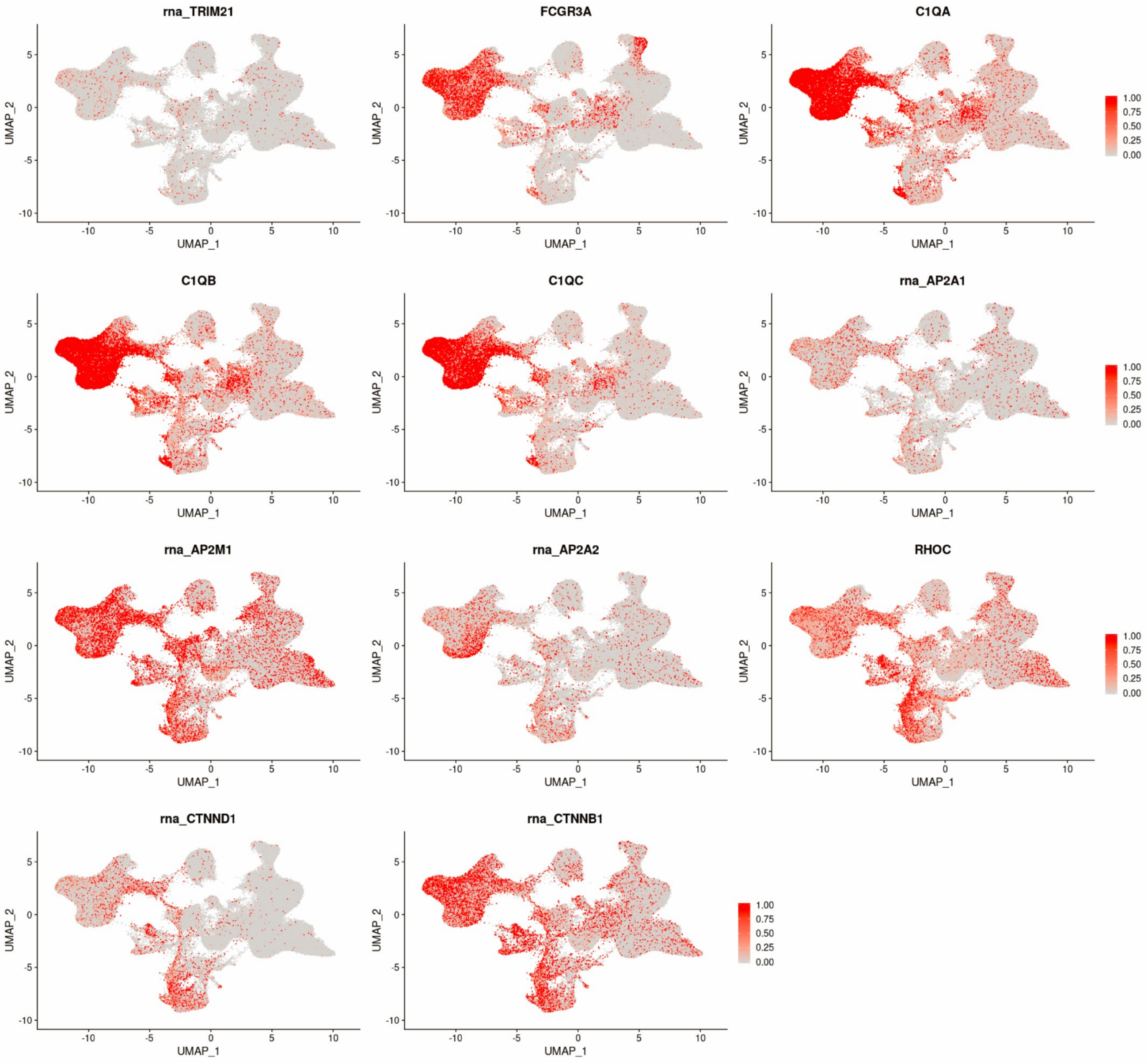

Fig. S5 Representative illustration of TRIM21, FCGR3A, C1QA, C1QB, C1QC, AP2A1, AP2M1, AP2A2, RHOC, CTTND1 and CTNNB1 expression. The expression of TRIM21, FCGR3A, C1QA, C1QB, C1QC, AP2A1, AP2M1, AP2A2, RHOC, CTTND1 and CTNNB1 were plotted.

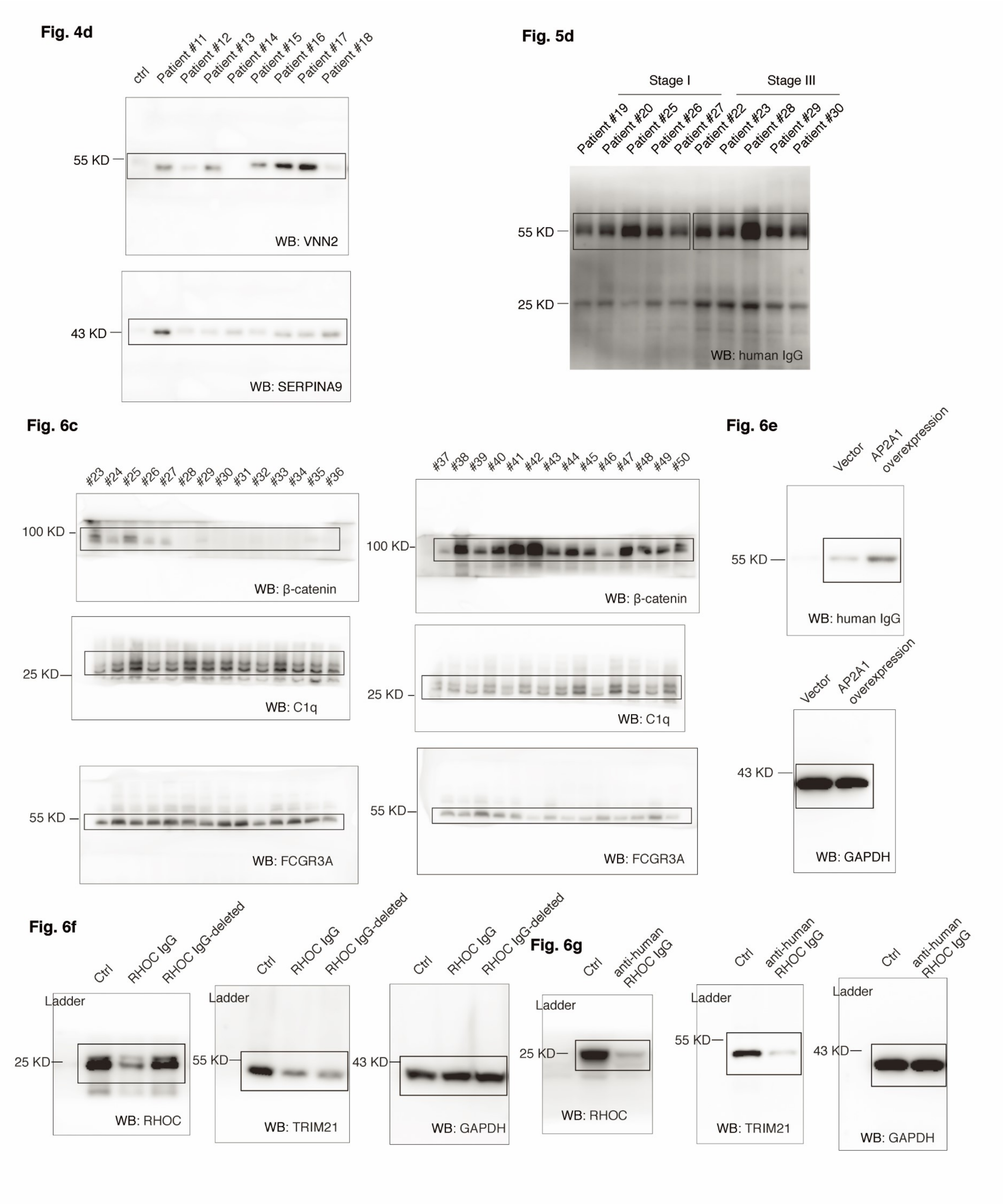

Fig. S6 Full blots of figures
