# Supplementary material for: Single-cell transcriptome and antigen-immunoglobin analysis reveals the diversity of B cells in non-small cell lung cancer
Source: Genome Biol. 2020 Jun 24;21:152. doi: 10.1186/s13059-020-02064-6 (PMC7315523; doi:10.1186/s13059-020-02064-6)
Supplement: Supplementary file 5 — Additional file 5. Review history. [file 13059_2020_2064_MOESM5_ESM.docx]

Review History

**First round of review**

**Reviewer 1**

**Are you able to assess all statistics in the manuscript, including the appropriateness of statistical tests used?** Yes, and I have assessed the statistics in my report.

**Comments to author:**

This study perform scRNAseq of mononuclear cells isolated from 6 patients with lung cancer (NSCLC). By comparison to PMBC, the authors identify two subtypes of B cells in tumors (regulatory-like and plasma-like). They show that their relative abundance varies according to tumor stage. They use supernatants/in vitro models to test the relevance of each cell type on the proliferation/phenotypes of lung cancer cell lines. While regulatory-like B cells seem to inhibit tumor growth, plasma-like B cells seem to have different effects on cancer cell line based on tumor stage. Mechanistic follow-ups suggest various pathways/mechanisms of action. Unfortunately, this reviewer (with expertise in cancer, genomics and single-cell analyses, but more limited expertise in immunology) thinks that the study suffers from major limitations.
Major issues:
- the number of patients is small (6), especially when analyzing multiple stages of disease;
- throughout the text, "significantly" is used extensively, but most of the time without a supporting statistical test;
- there is no description at all of the computational methods for single-cell analysis (other than citing a pipeline); it is unclear if any QC metric was used; any filtering step to remove low QC, filter doublets etc; identifying many clusters (the authors claim 23) can be technical; the color code for the tsne in fig1 is not explained; the origin of PBMC is not explained (matched patients to NSCLC?) etc; overall the lack of details precludes any valid conclusion/assessment of data;
- this issue is exemplified by the extreme approximation of some of the founding analysis of the manuscript: in the result section, on page 5, the authors claim "the transcriptome of CD79A+CD20+ was somehow similar to regulatory B cells... we thus defined this subsets as regulatory-like". The same is said for transcriptome of CD79A+CD20- cells being somehow similar to those of plasma B cells. In these statements, no comprehensive comparison is performed; no statistical test for similarity is offered; the conclusion/interpretations thus appear very weak at best;
- the manuscript's main quality is probably in the analysis of a 30 patient cohort by flow/IHC and the analysis of survival for markers of B cells subsets;
- the mechanistic follow-ups (many of them) are unfortunately not very well executed; "control" is most of the time not defined/explained; the impact of the various manipulations on in vitro cell lines is frequently modest and without further defining the baseline/control, one wonders about the reproducibility/relevance of such small deviations from baseline; the directions followed are very disparate and each one of them is followed in a questionable/shallow manner; the authors would be better of focusing on one mechanistic angle and providing much stronger support for their (far too many) claims.

**Reviewer 2**

**Are you able to assess all statistics in the manuscript, including the appropriateness of statistical tests used?** There are no statistics in the manuscript.

**Comments to author:**

In this manuscript by Chen et al., authors analyzed B-cell clusters from six lung cancer patients by single cell RNA sequencing, and reported IgG-producing B cells and regulatory-like B cells. Based on B cell type classification and the molecular markers such as CTNNB1, AP-2 complex, C1QA and TRIM21, authors demonstrated the function of two B cell subtypes in tumor growth of lung cancer by co-culture with lung cancer cell lines. Authors showed direct effect by coculture with regulatory-like B cells or transferring culture supernatant to lung cancer cell lines. In case of IgG-producing B cells, functional study was done in reconstituted system, too. However, stromal and immune microenvironments of tumor tissue are working together in tissue remodeling. In this sense, authors should show overall cellular landscape and receptor-ligand interaction in lung cancer tissue as well.

Major points
1.     Authors analyzed single cells of lung cancer from six patients, and emphasized IgG-producing B cells and regulatory-like B cells. However, there are macrophage, stroma cell, T cells and tumor cell fractions in patient tumor tissue. Using CellPhoneDB, authors can simulate the cellular network between different cell population based on receptor-ligand interactions.

2.     Cell population analysis on exhausted T cells is most important to understand immune status of lung cancer. Tumor mutation burden and PD-1/PD-L1 expression are also critical to determine the clinical outcomes to immunotherapy. Is there any correlation between those parameters and B cell status?

3.     Functional studies on CTNNB1, AP-2 complex, C1QA and TRIM21 in Figure 4-7 demonstrated the tumor-promoting activity of IgG-producing B cells. The candidate proteins were found by proteomic analysis, and the result was summarized in Supplementary Table S3 "Targets of pathology antibodies in NSCLC tumors". Authors should disclose the criteria to select those proteins in functional study with statistical analysis on proteomic analysis data.

4.     In Figure 4, regulatory-like B cells could directly suppress the tumor growth. What is the mechanism?    


Minor points
1.     In Figure 3, authors showed the expression of VNN2 and SERPINA9 in regulatory-like B cell from three patients, Patient #3, #5 and #8 with different stages. In the other figures, authors showed the differential expression pattern in five to 32 patients in early and late stages. Please check the expression of VNN2 and SERPINA9 in more cases for the validation.
2.     Please describe the method to divide groups with the expression levels of CD79A and CD20 for Figure 2F.
3.     What is CCK8 assay in Figure 3?

**Authors Response**

**Point-by-point responses to the reviewers’ comments:**

Reviewer #1: This study perform scRNAseq of mononuclear cells isolated from 6 patients with lung cancer (NSCLC). By comparison to PMBC, the authors identify two subtypes of B cells in tumors (regulatory-like and plasma-like). They show that their relative abundance varies according to tumor stage. They use supernatants/in vitro

models to test the relevance of each cell type on the proliferation/phenotypes of lung cell lines. While regulatory-like B cells seem to inhibit tumor growth, plasma-like B cells seem to have different effects on cancer cell line based on tumor stage. Mechanistic follow-ups suggest various pathways/mechanisms of action. Unfortunately, this reviewer (with expertise in cancer, genomics and single-cell analyses, but more limited expertise in immunology) thinks that the study suffers from major limitations.

*We appreciate the reviewer’s comments and suggestions. As suggested, we added 5 more scRNA-seq analysis and a total of 11 fresh tumor tissues were analyzed, which includes 115,545 single cells in the revised manuscript. As suggested, we have re-organized our results to make our data clearer. We also systematically analyzed the cell-type-specific gene in the prognosis of the NSCLC and highlighted the prognostic value of B cells. We also demonstrated a tumor-suppressive function of the naïve-like B cells and bi-functional plasma-like B cells in different stages of NSCLC.*

Major issues:

- the number of patients is small (6), especially when analyzing multiple stages of disease;

***Response****: We appreciated the reviewer for pointing out the problems of the sample size. We have now added 5 single-cell transcriptomic analysis in the revised manuscript. A total of 11 scRNA-seq datasets (including 5 patients with stage I NSCLC and 6 patients with stage III NSCLC) and 115,545 single cells were analyzed in the revised manuscript. The data looks much better when we used 11 samples to analyze, but the conclusion did not change. Moreover, the major findings from the scRNA-seq analysis were further validated by using two independent cohort, including 30 primary samples with Flow cytometry analysis and 164 primary samples with IHC staining. Also, we studied the function of the B cells using samples from a 46-patient cohort, including eight tissue samples used for testing the function of the naïve-like B cells, six tissue samples used for testing the function of plasma-like B cells, 32 tissue samples used for identifying the targets of plasma-like B cells produced IgG.*

- throughout the text, "significantly" is used extensively, but most of the time without a supporting statistical test;

***Response****: We really thank the reviewer for pointing out the overused word*  “*significantly” in our previous manuscript. We have now carefully used this word only in the results with statistical support. And the "significantly" was changed into a more accurate description such as the fold change and p-value or deleted when we reorganized our manuscript.*

*The “Moreover, we observed that the regulatory-like B cells but not the plasma-like B cells was significantly decreased at the advanced stages of lung cancers” (Lines 138-139) were change into “In addition, we observed a decrease of the CD20^+^CD79^+^ B cell in the advanced stages of NSCLC (Figure 3D).”.*

*“The regulatory-like B cells significantly inhibited the growth of A549 and H1299 cells” (Lines 156-157) changed into “The co-culture with CD20^+^ B cells significantly inhibited the growth of A549 and H1299 cells (p<0.001) (Figure 4A and Figure S3A).”*

*“We found that overexpression of SERPINA9 and VNN2 significantly inhibit the growth of A549 and H1299.” changed into “We found that the overexpression of SERPINA9 and VNN2 inhibited the growth of A549 and H1299 cells (Figure 4E and Figure S3C).”*

*“The plasma-like B cells from stage III tumors significantly promote proliferation of A549 cells”(Lines 183-184) change into “we found that the plasma-like B cells from stage III promote proliferation of A549 cells.”*

*“while the IgG isolated from stage III NSCLC tumor significantly accelerated the growth of A549 cells” (Lines 195-196) change into “while the culture supernatants of plasma-like B cells isolated from stage III promote the cell growth of A549 and H1299 cells”*

- there is no description at all of the computational methods for single-cell analysis (other than citing a pipeline); it is unclear if any QC metric was used; any filtering step to remove low QC, filter doublets etc; identifying many clusters (the authors claim 23) can be technical; the color code for the tsne in fig1 is not explained; the origin of PBMC is not explained (matched patients to NSCLC?) etc; overall the lack of details precludes any valid conclusion/assessment of data;

***Response****: We appreciated the reviewer for pointing out the inadequate description of the single-cell RNA-seq analysis. The raw sequences were processed with Cell Ranger (version 3.0.2), a package that takes Illumina bcl or fastq files as input and generates a count matrix after filtered errors and biases. Filtered reads were aligned to the human genome (GRCh38) with STAR[1], a high-performance community-standard aligner. After alignment, reads were translated into a UMI matrix. The matrix of read counts per gene per sample was further analyzed by the Seurat suite (version 3.1.0)[2] in the R program (version 3.6.0). A total of 144,271 cells that passed quality control steps implemented in Cell Ranger were obtained. We further applied a quality-control to get highly reliable cells, cells meeting any of the following criteria were excluded: <500 or >6,000 unique genes expressed, >40,000. UMIs, or >50% of reads mapping to mitochondria. These steps removed an additional 28,726 cells, resulting in a final dataset of 115,545 cells. We quantified gene expression across 35,538 genes, of which 19,540 were expressed in at least one cell.*

*We agree with the reviewer that the clustering resolution is a subjective parameter that could be varied quantitatively as a function of the number of cells being clustered, but in general it is difficult to choose an appropriate resolution in an automated fashion.*

*To keep a standard procedure for clustering, we used a value of 0.5 for the resolution.*

*To investigate transcriptional heterogeneity and to undertake initial cell clustering, we applied a dimensionality reduction with Principal Component Analysis (PCA). We selected the top 30 Principal Components (PCs) that explained more variability than expected by chance using a permutation-based test in Seurat. For the cell clustering within primary clusters (sub-clustering), we selected variable numbers of PCs for dimensionality reduction using either permutation-based analyses or heuristic methods in Seurat. We used PC loadings as input for a graph-based approach to cluster cells by cell type and as input for uniform manifold approximation and projection (UMAP) for reduction to two dimensions for visualization purposes.*

*Also, we apologize for the missing description of the color code, the cell types of each color were added alongside the cluster name. We apologized for the unclear description of the PBMC single cell RNA-seq datasets. The PBMC scRNA-seq datasets were obtained from the database of 10X genomics (https://support.10xgenomics.com/single-cell-gene-expression/datasets). The clusters expressing CD79A or MS4A1 were identified as B cells. Cell barcodes of the B cell clusters and corresponding gene counts were extracted for generating B cell expression matrix. These datasets were used as a control for identification of the differences between naïve-like B cells and plasma-like B cells.*

*In the revised manuscript, we have included a detailed pipeline we used in the single-cell RNA-seq analysis. Also, the missing statement such as the color code for the UMAP, the origin of PBMC has been added in the revised manuscript.*

- this issue is exemplified by the extreme approximation of some of the founding analysis of the manuscript: in the result section, on page 5, the authors claim "the transcriptome of CD79A+CD20+ was somehow similar to regulatory B cells... we thus defined this subsets as regulatory-like". The same is said for transcriptome of CD79A+CD20- cells being somehow similar to those of plasma B cells. In these statements, no comprehensive comparison is performed; no statistical test for similarity is offered; the conclusion/interpretations thus appear very weak at best;

***Response****: We agree with the reviewer that a comprehensive comparison and statistical test for similarity were needed for the definition of the naïve-like B cells and Plasma-like B cells. We apologize for the unclear description of the method used in our previous manuscript. The Cluster specific genes were acquired using the FindMarkers algorithm in the Seurat suite. Test used for cell marker identification was ROC analysis. Cell types of each cluster were determined by scMatch[3] and manually checked with known cell surface markers.*

- the manuscript's main quality is probably in the analysis of a 30 patient cohort by flow/IHC and the analysis of survival for markers of B cells subsets;

***Response****: We appreciate the reviewer's comment. Indeed, the IHC results showing the CD20+CD79A+ B cells are associated with favorable clinical outcome of NSCLC. We apologized for the unclear description of the cohort we used in our study. We analyzed patients from four independent cohorts in different experiments. We analyzed single-cell RNA-seq datasets in an 11-patient cohort. We performed flow cytometry in a 30-patient cohort. We analyzed the co-exitance of naïve-like and plasma-like B cells in a 164-patient cohort. We studied the function of the B cells using samples from a 46-patient cohort, including eight tissue samples used for testing the function of the naïve-like B cells, six tissue samples used for testing the function of plasma-like B cells, 32 tissue samples used for identifying the targets of plasma-like B cells produced IgG. The survival for markers of B cells subsets were also validated in the TCGA cohort. We also apologize for the unclear description of our results. We have now rewritten the manuscript to a better illustration of our results.*

- the mechanistic follow-ups (many of them) are unfortunately not very well executed; "control" is most of the time not defined/explained; the impact of the various manipulations on in vitro cell lines is frequently modest and without further defining the baseline/control, one wonders about the reproducibility/relevance of such small deviations from baseline; the directions followed are very disparate and each one of them is followed in questionable/shallow manner; the authors would be better of focusing on one mechanistic angle and providing much stronger support for their (far too many) claims.

*Response: We apologized for the unclear description of the mechanistic follow-ups. After identifying the two type of B cells in the microenvironment of NSCLC, we determined the function and underlying of these B cells in the tumor progression of NSCLC. We demonstrated that the naïve-like B cells inhibit cell proliferation of the NSCLC cells via production of secreted factors such as VNN2 and SERPINA9. The Plasma-like B cells exert different function in stage I and stage III NSCLC, it inhibited the cell growth in stage I NSCLC but promote cell growth in stage III NSCLC. The diverse function of the plasma-like B cells was attributed by the production of antibodies. We further determined the characteristics of the targets of the IgGs produced by the newly identified plasma-like B cells. We have now reorganized this part of results. We apologized for the unclear description of the “control”, we have now included the description of our control in the revised manuscript.*

*We really appreciated the reviewer’s suggestion that it would be better of focusing on one mechanistic angle and providing much stronger support. In the revised manuscript, we reorganized the last three-part of the results and shorten the observations on the diverse function of the antibodies. We conclude that the IgG produced by the plasma-like B cells could be imported into the tumor cells via the AP2 protein complex and degraded the targets of the IgGs in a TRIM21-dependent ubiquitin pathway.*

Reviewer #2: In this manuscript by Chen et al., authors analyzed B-cell clusters from six lung cancer patients by single cell RNA sequencing, and reported IgG-producing B cells and regulatory-like B cells. Based on B cell type classification and the molecular markers such as CTNNB1, AP-2 complex, C1QA and TRIM21, authors demonstrated the function of two B cell subtypes in tumor growth of lung cancer by co-culture with lung cancer cell lines. Authors showed direct effect by coculture with regulatory-like B cells or transferring culture supernatant to lung cancer cell lines. In case of IgG-producing B cells, functional study was done in reconstituted system, too. However, stromal and immune microenvironments of tumor tissue are working together in tissue remodeling. In this sense, authors should show overall cellular landscape and receptor-ligand interaction in lung cancer tissue as well.

*We appreciate the reviewer’s comments and suggestions. As suggested, we added analysis on the interactions between B cells and other cells in our scRNA-seq. Indeed, despite the secreted proteins of the B cells affect the tumor cells, we also found that the B cells could interact with some of the cells in the microenvironment. Moreover, we added 5 more scRNA-seq analysis, and a total of 11 scRNA-seq and 115,545 single cells were used in the revised manuscript.*

*We identified six classes of T cells (C0, C1, C3, C11, C13, C21) (Figure 1B, Figure S1 and Table S1). The C0 and C11 cluster of T cells are with high expression level of CD3E, CD8A and CD8B, suggesting CD8^+^ T cells; the C13 cluster of T cells expressed high level of NCAM1 (CD56), CD3E, NKG7, and low level of CD8A and CD8B, suggesting possible natural killer T (NKT) cells; the C1 cluster expressed CD4 genes, indicating CD4^+^ T cells; the C21 expressed high levels of CD4 and GZMB, indicating CD4^+^ CTLs; The C3 cluster is with high expression of FOXP3 and IL2RA (CD25), which are possible Tregs. We identified two classes of B cell (C4, C6), which expressed CD79A (in C4 and C6) and MS4A1 (CD20) (in C4). We identified five classes of tumor cells (C8, C9, C10, C18, C20), which expressed the tumor cell marker genes EPCAM, CEACAM6 or MKI67. We also identified five classes of monocytes (C2, C5, C7, C12, C14), expressing CD14 and CD86. The C2, C5, C7 and C14 expressed high levels of CD163 and MRC1 (CD206), suggesting the majority monocytes/macrophages in the TME are M2-polarization macrophage. We also observed red blood cell (C19) expressing HBA1 and HBB, and epidermal or vascular cells (C15 and C17) expressing COL3A1 and COL1A2 (in C15) or IGFBP7 (in C17), and mast cells (C16) expressing TPSAB1 and TPSB2 in the scRNA-seq (Figure 1C, Figure S1 and Table S1). We next detected the ratio of 22 types of cells in different patients. In accordance with previous observations[4], the composition of each cell types was largely different across the 11 tumor samples (Figure 1D).*

Major points:

1. Authors analyzed single cells of lung cancer from six patients, and emphasized IgG-producing B cells and regulatory-like B cells. However, there are macrophage, stroma cell, T cells and tumor cell fractions in patient tumor tissue. Using CellPhoneDB, authors can simulate the cellular network between different cell population based on receptor-ligand interactions.

***Response****: We really appreciated the reviewer’s suggestion. As suggested, we performed the receptor-ligand interaction to further dissect the interaction between B cells and other cells in the microenvironment of NSCLC using the CellPhoneDB* [5]*. We identified a cell-cell interaction network among the two cell types (and other cell types).*

*It is noteworthy that the genes expressed by the B cells showed a strong interaction with other immunes cell types (Figure S2), suggesting an essential role of B cells in the microenvironment of NSCLC. We have now added these results as supplemental Figure 2 in our revised manuscript. We also performed the cell-cell interaction analysis to further dissect the correlation between T cells and B cell status. We performed an integrative analysis using the targets of B cell-secreted antibodies and the cell-cell interaction-based receptor-ligand and found the B cells secrete antibodies against the COPA which interact with CD74 which potentially involved in the tumor cell-macrophage interaction, suggesting a potential role of the B cell-secreted protein in the regulation of cell-cell interaction in the microenvironment of NSCLC.*

2. Cell population analysis on exhausted T cells is most important to understand immune status of lung cancer. Tumor mutation burden and PD-1/PD-L1 expression are also critical to determine the clinical outcomes to immunotherapy. Is there any correlation between those parameters and B cell status?

***Response****: As suggested by the reviewer, we checked the correlation between naïve-like B marker MS4A1 (CD20) and plasma-like B marker (BCMA) with PDCD1 (PD-1), CD274 (PD-L1) and the TMB (tumor mutation burden) in the TCGA cohort. We found that the expression of CD20 were positively correlated with the expression of PDCD1 (r=0.56, p=7.14e-43) and CD274 (r=0.26, p=1.96e-9), and TMB (r=0.45, p=1.96e- 29), the expression of BCMA were also were positively correlated with the expression of PDCD1 (r=0.44, p=8.87e-28) and CD274 (r=0.19, p=2.245e-5), and TMN (r=0.32,*

*p=4.67e-17), suggesting the co-exitance of high levels of the tumor mutation burden, high level of PDCD1 expression and the infiltration of naïve-like B cells. We have now added this data in revised Figure 3.*

3. Functional studies on CTNNB1, AP-2 complex, C1QA and TRIM21 in Figure 4-7 demonstrated the tumor-promoting activity of IgG-producing B cells. The candidate proteins were found by proteomic analysis, and the result was summarized in Supplementary Table S3 "Targets of pathology antibodies in NSCLC tumors". Authors should disclose the criteria to select those proteins in functional study with statistical analysis on proteomic analysis data.

*Response: We apologized for the unclear description of our results. This important comment was similar with the one raised by reviewer 1. We have now shortened this part to make our results clear. The criteria to select those proteins were based on the Gene ontology of these targets and the occurrence of the proteins identified by proteinomics. We classified the targets of the antibodies into three clusters. The IgGs, the Fc-binding proteins and the targets of the antibodies. For the Fc-binding part, we demonstrated that some Fc-binding proteins were mainly expressed in the macrophages, but the scRNA-seq dataset suggesting the macrophage in the NSCLC are M2-type which lack the tumor suppressive function. We also found the interaction between AP2-complex and TRIM21 via Fc-binding compacity, we demonstrated that the interaction with AP2 complex was responsible for the transportation of the antibodies into tumor cells and the interaction with TRIM21 contributed to antibody-depended ubiquitin degradation. For the target part, we demonstrated that the antibodies against the RHOC were responsible for the degradation of RHOC protein in the tumor cells in AP2-TRIM21 dependent manner.*

4. In Figure 4, regulatory-like B cells could directly suppress the tumor growth. What is the mechanism?

*Response: We apologized for the unclear description of our mechanistic studies. To explain the mechanisms underlying the regulatory-like B cells (now corrected as naïve-like B cells in the revised manuscript) in suppressing tumor growth, we determined the expression pattern of these B cells in our single-cell transcriptomic studies and identified many secretion proteins. We further demonstrated that the secreted protein, such as VNN2 and SERPINA9, contributes to the suppressive function of B cells on the tumor cells.*

Minor points:

1. In Figure 3, authors showed the expression of VNN2 and SERPINA9 in regulatory-like B cell from three patients, Patient #3, #5 and #8 with different stages. In the other figures, authors showed the differential expression pattern in five to 32 patients in early and late stages. Please check the expression of VNN2 and SERPINA9 in more cases for the validation.

***Response****: As suggested, we checked the expression of VNN2 and SERPINB9 in the culture supernatant of the naïve-like B cells in more patients. And a total of 8 samples were detected. Also, we apologize for the unclear description of the experiments, due to the requirement of a large number of cells for detection, samples could only be used in either functional studies, mechanism exploration or scRNA-seq. A total of 240 tissues were used in different experiments.*

2. Please describe the method to divide groups with the expression levels of CD79A and CD20 for Figure 2F.

***Response****: As suggested, we have added the method to divide groups with the expression levels of CD79A and CD20 in the revised manuscript. Scoring of immunohistochemistry: All immunostained slides and matching hematoxylin and eosin–stained sections were scanned with the MoticEasyScan digital scanner. The TILs were composed of mononuclear cells, including lymphocytes, macrophages and plasma cells. Intra-alveolar macrophages were not considered as part of the immune infiltration [6]. All IHC results were independently scored by two pathologists (Likun Hou and Chunyan Wu). In case of disagreement, the slides were re-examined and the observers reached a consensus. A semiquantitative manual scoring was used to*

*evaluate the percentage of TILs exhibiting membrane staining (tumor proportion score). The overall infiltration of the naïve-like B cells was quantified by multiplication of the the ratio of CD20+CD79+ double positive cells among the TILs cells. We defined the ratio*>*10% as naïve-like B^high^ cells, while the ratio*≤*10% as naïve-like B^low^ cells..*

3. What is CCK8 assay in Figure 3?

***Response****: We apologized for the unclear description of our results. We used the CCK8 assay to determine the effects of B cells as well as its secreted proteins on the viability of tumor cells. To dissect the direct effects of the B cells on the tumor cells, we co-cultured the B cells with the NSCLC cell line. After 48 hours of co-culture, B cell-containing culture media was removed and replaced with fresh culture media and cultured for further 4 hours and the cell viability of A549 and H1299 cells were determined by CCK8 assays. We have now included a detailed description of this assay in the revised manuscript.*

1. Dobin A, Davis CA, Schlesinger F, Drenkow J, Zaleski C, Jha S, Batut P, Chaisson M, Gingeras TR: **STAR: ultrafast universal RNA-seq aligner.** *Bioinformatics* 2013, **29:**15-21.

2. Stuart T, Butler A, Hoffman P, Hafemeister C, Papalexi E, Mauck WM, 3rd, Hao Y, Stoeckius M, Smibert P, Satija R: **Comprehensive Integration of Single-Cell Data.***Cell* 2019, **177:**1888-1902 e1821.

3. Hou R, Denisenko E, Forrest ARR: **scMatch: a single-cell gene expression profile annotation tool using reference datasets.** *Bioinformatics* 2019, **35:**4688-4695.

4. Azizi E, Carr AJ, Plitas G, Cornish AE, Konopacki C, Prabhakaran S, Nainys J, Wu K, Kiseliovas V, Setty M, et al: **Single-Cell Map of Diverse Immune Phenotypes in the Breast Tumor Microenvironment.** *Cell* 2018, **174:**1293-1308 e1236.

5. Efremova M, Vento-Tormo M, Teichmann SA, Vento-Tormo R: **CellPhoneDB:**

**inferring cell-cell communication from combined expression of multi-subunit**  **ligand-receptor complexes.** *Nat Protoc* 2020.

6. Al-Shibli KI, Donnem T, Al-Saad S, Persson M, Bremnes RM, Busund LT: **Prognostic effect of epithelial and stromal lymphocyte infiltration in non-small cell lung cancer.** *Clin Cancer Res* 2008, **14:**5220-5227.

**Second round of review**

**Reviewer 1**

My concerns have been addressed by the revisions and the dataset is much expanded and improved.

**Reviewer 2**

Questions and comments were answered with additional experiments and analysis. Tertiary lymphoid structure (TLS) in lung cancer plays an important role in tumor immunity. This manuscript will provide insights on the function of TLS in lung cancer immunology.
